# Cationically Modified PVA-Based Electrospun Nanofiber Membrane for Adsorptive PFAS Removal from Water

**DOI:** 10.1021/acsaenm.5c00822

**Published:** 2025-12-16

**Authors:** Md. Nahid Pervez, Tao Jiang, Boyu Li, Behnia Bitaraf, Aswin Kumar Ilango, Marina Maria Ioanniti, Caroline Schaeffer, Haralabos Efstathiadis, Mehmet V. Yigit, Yanna Liang

**Affiliations:** † Department of Environmental and Sustainable Engineering, 1084 University at Albany, State University of New York, Albany, New York 12222, United States; ‡ Department of Nanoscale Science and Engineering, 1084 University at Albany, State University of New York, Albany, New York 12222, United States; § Department of Chemistry, 1084 University at Albany, State University of New York, Albany, New York 12222, United States

**Keywords:** PFAS, Electrospinning, Surfactant, Stormwater, Nanofiber membrane

## Abstract

Per- and polyfluoroalkyl substances (PFAS), a diverse range of anthropogenic organic compounds, pose significant concerns to society due to their potential harmful impacts on human health and ecosystems. While there are other methods for removing PFAS from water, adsorption remains a viable and efficient option. The present research reports an adsorptive nanofiber membrane prepared through electrospinning in the presence of poly­(vinyl alcohol) (PVA) and a cationic surfactant, cetyltrimethylammonium chloride (CTAC), blended solution. This modified PVA membrane was observed to achieve nearly 100% capture of all 10 target PFAS, each at 10 μg/L in deionized water. The pseudo-second-order model most accurately represented the adsorption kinetics, characterized by rapid adsorption (within 60 s). The Toth isotherm model effectively fitted the isotherm data, indicating that the adsorption of PFAS onto the membrane involved complex interactions. The hypothesized adsorption mechanisms, including electrostatic and hydrophobic interactions, were validated through detailed adsorption kinetics, isotherms, thermodynamic analyses, and physicochemical characterization. Remarkably, the performance of the modified system remained unaffected by variations in solution pH and natural organic matter, while being slightly affected by ionic strength, with 90–100% removal effectiveness of PFAS in stormwater. This work highlights the significance of electrospun nanofiber membrane-based adsorbents for the efficient removal of PFAS from real water.

## Graphical abstract

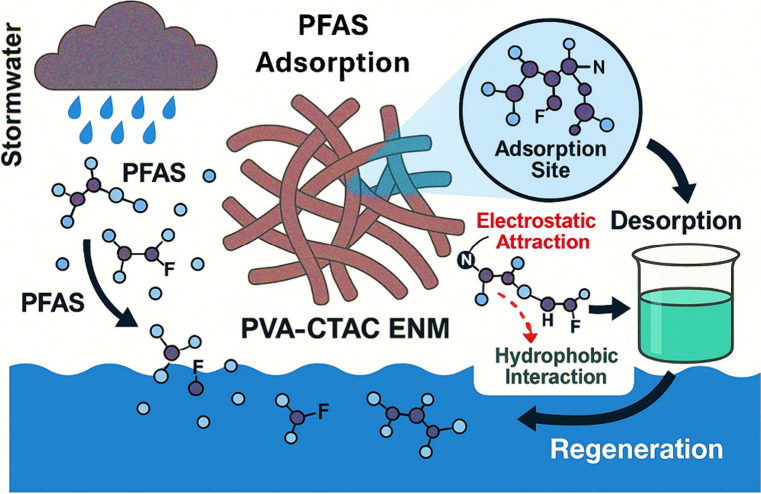

## 1 Introduction

Per- and polyfluoroalkyl substances (PFAS) are a diverse group of synthetic organic molecules characterized by the substitution of hydrogen atoms in their carbon backbones with fluorine, either whole or partially.^1,2^ PFAS are widely used in numerous industrial and consumer applications due to their remarkable chemical stability, surfactant properties, and resistance to environmental degradation.^3,4^ PFAS, produced for over 60 years and now widely found in the environment, have raised significant concerns from the general public and the regulatory agencies due to their potential risks to human health and the ecosystem.^5^ Consequently, it is imperative to swiftly implement actions to eliminate PFAS from polluted environments.

To remove PFAS from contaminated water, capture followed by destruction has been widely accepted as the most cost-effective approach. During the capture phase, an adsorbent is required to selectively attract PFAS away from water to the adsorbent itself.^6,7^ The spent PFAS-laden adsorbent can then be subject to regeneration/reuse or destruction of PFAS.^8,9^ To selectively remove PFAS from water, a wide range of adsorbents has been reported in the literature, including carbon,^10^ ion-exchange resins,^11^ biosorbents,^12^ and clay.^13^

Adsorptive nanofibrous membranes have been explored in recent years, offering a potential solution to reduce energy consumption and mitigate fouling in wastewater treatment applications, thanks to their high surface-to-volume ratio and variable surface functionalization.^14,15^ Electrospinning is a straightforward technique to produce nanofibrous membranes with diameters usually between 100 and 1000 nm, which are typically 2 to 3 orders of magnitude smaller than conventional fibers.^16^ A distinct thinning process known as the ″bending (or whipping) instability″ distinguishes electrospinning from traditional fiber spinning methods, including wet, dry, and melt spinning. Electrospinning is powered by electrical current rather than mechanical force. A “Taylor cone” is formed when the droplet of an electrospinning polymer solution at the spinneret’s tip is exposed to an electric field. Electrospinning starts with the ejection of a jet of polymer solution from the tip of the Taylor cone when the applied electrical force is greater than the surface tension of the solution.^17^ Subsequently, the polymer solution jet thins out and curves in a spiral pattern as the loop’s diameter increases. The process culminates in a nonwoven membrane deposit on a grounded metal collector, which is formed when the polymer solution jet hardens due to rapid solvent evaporation.^18^

Recent research has shown that electrospun nanofibrous membranes (ENM) with anion-exchange amine side chains and a porous structure may effectively adsorb PFAS at very low pressure conditions.^19^ Through a combination of electrostatic and hydrophobic interactions, these membranes were able to adsorb over 90% of PFOA molecules,^20^ while desorption under alkaline/methanol conditions resulted in a 35-fold enrichment of PFOA.^21^ In particular, Dai et al.^22^ synthesized an ENM by integrating multiwalled carbon nanotubes (MWCNTs) with poly­(d,l-lactide) in an electrospinning solution. The ENM’s specific surface area, adsorption rate, and PFOS adsorption capacity were all enhanced by the addition of MWCNTs (16.29 ± 0.26 μmol/g). However, ENM adsorbents still face two critical hurdles that limit their practical use for PFAS remediation. First, removal performance drops in realistic mixtures where short- and long-chain congeners compete for sites, exposing chain-length–dependent trade-offs seldom revealed in single-solute tests. Second, common cocontaminants such as natural organic matter, anions, and trace metals can foul or recharge surfaces, sharply reducing capacity and selectivity. These gaps underscore the need for ENMs with orthogonal binding domains and water-resistant surfaces, rigorously vetted under chemically complex conditions to ensure broad-spectrum, field-relevant PFAS remediation.

Here, we report a simplified procedure for synthesizing novel ENM adsorbents by modifying poly­(vinyl alcohol) (PVA) with polyethylenimine (PEI), chitosan, or a cationic surfactant (cetyltrimethylammonium chloride, CTAC) and evaluate their ability to remove a mixture of PFAS from pure water. In line with results from previous studies,^13,23,24^ PVA modified by CTAC demonstrated much higher removal of anionic PFAS than those modified by PEI or chitosan. Also, the modification may effectively stabilize PVA by physically attaching it with surfactant molecules without causing oxidation or defects, maintaining its integrity, lowering surface tension, and increasing the viscosity and conductivity.^25^ In this work, the PVA-CTAC material functioned solely as an adsorbent rather than a filtration membrane; the term membrane is used only to describe its electrospun fibrous structure. Our study thus focused on the design and synthesis of PVA-CTAC ENM with two distinct characteristics: (1) smaller diameter with positive surface charges and (2) regenerability and reusability obtained through the cross-linking approach. Specifically, this study aimed to investigate how surfactant modification influenced PFAS adsorption by ENM comprehensively. By systematically comparing PVA and PVA-CTAC ENM, we sought to elucidate the roles of CTAC doping, diameter, and surface charge in capturing both long- and short-chain PFAS in water with different physicochemical properties. In addition, given the unique capability of the PVA-CTAC ENM in capturing short chain PFAS as detailed below, it was tested on the removal of PFAS in stormwater (SW). To our knowledge, this study represents the first exploration of this particular material specifically for PFAS adsorption and particularly for capturing short chain PFAS in SW.

## 2 Experimental Section

### 2.1 Chemical Reagents and Materials

The chemical reagents and the physicochemical properties of PFAS employed in this research are detailed in Tables S1 and S2, respectively. We collected a sample of stormwater in March 2025 from the University at Albany, State University of New York’s campus, and stored it at 4 °C. Table S3 details the stormwater’s composition. Throughout the experiment, working solutions were prepared using deionized (DI) water, which has a resistivity of at least 18.2 MΩ·cm.

### 2.2 PVA-CTAC Electrospun Nanofiber Membrane Preparation

The PVA-CTAC nanofiber membranes were made in the following manner. Briefly, 50 mL of DI water was added with 10 wt % PVA and 0, 10, 20, 30, or 40 wt % CTAC. The mixture was then magnetically agitated for 4 h at 80 °C to ensure even mixing and was allowed to cool to room temperature thereafter. An electrospinning apparatus (NS plus Inovenso Ltd., Turkey) was subsequently used for all electrospinning investigations. Using a 21-gauge stainless steel needle, the mixed solution was inserted into a 10 mL syringe. A 25 kV high potential voltage was used with a 0.5 mL h^–1^ solution flow rate. The PVA-CTAC nanofibers were collected using aluminum foil, which was held 10 cm from the needle tip. After removing the nanofiber substrate from the aluminum foil, it was vacuum-dried at 60 °C for 24 h. Then, for 30 min, the electrospun membrane was submerged in a cross-linking solution consisting of 96 mL of acetic acid, 4 mL of glutaraldehyde (GA), and 0.1 mL of concentrated HCl.^19^ This was followed by a complete rinsing with DI water and storage of the cross-linked membrane at room temperature prior to application. Additionally, the synthesis of PVA–PEI and PVA-chitosan ENMs is described in Text S1 and Text S2.

### 2.3 Adsorption Experiments

The adsorption studies were conducted in a batch mode using 50 mL polypropylene centrifuge tubes from Corning Inc. (Corning, NY, USA). All tests were performed with three replicates. Each tube was supplemented with a mixture of eight perfluoroalkyl acids (PFAAs), namely C6–C10 perfluorocarboxylic acids (PFCAs), and C4, C6, C8 perfluorosulfonic acids (PFSAs); as well as two alternatives to perfluorooctanoic acid (PFOA) and perfluorooctanesulfonic acid (PFOS), namely GenX and 6:2 fluorotelomer sulfonic acid (6:2 FTSA), respectively. The starting concentration of each PFAS was 10 μg/L. Before adding an adsorbent at 100 mg/L, 500 μL of the PFAS solution was removed from each tube and used as an initial sample (0 min). Once the adsorbent was added, the tubes were put on a rotating shaker set at 150 rpm. Samples were collected from each tube at 1, 2, 8, and 24 h. Following centrifugation at 14,000 g for 10 min, the supernatant was analyzed for concentration of each PFAS using an Agilent Technologies 1290 Infinity II LC system coupled with a 6470 Triple Quad Mass Spectrometer (LC-MS/MS, Santa Clara, CA, USA). The effectiveness of the adsorbents in removing PFAS by adsorption was assessed using eq 1 in the following manner:

1 $$\mathrm{Removal efficiency}\left(\%\right)=\frac{C_{i}-C_{t}}{C_{i}}\times 100$$

where C_i_ and C_t_ are the PFAS concentration at initial and time (t), respectively.

### 2.4 Adsorption Kinetics, Isotherms, and Thermodynamic Studies

Adsorption kinetic experiments were conducted using an initial concentration of 10 μg/L for each of the ten PFAS at 25 °C and 150 rpm, with an adsorbent dose of 100 mg/L and a pH of 6.8 for various contact times (5, 15, 30, 60, 90, 120, 180, and 240 min). The adsorption kinetics data were further analyzed and represented using three commonly used kinetic models: pseudo-first order (PFO), pseudo-second order (PSO), and intraparticle diffusion (IPD)^26^ with these three equations:

2 $$\mathrm{PFO}:q_{t}=q_{e}\left(1-e^{{-k}_{1}t}\right)$$

3 $$\mathrm{PSO}:q_{t}=k_{2}q_{e}^{2}t/\left(1+k_{2}q_{e}t\right)$$

4 $$\mathrm{IPD}:q_{t}=k_{d}t^{1/2}+C_{d}$$

The variables t (min), q_t_ (mg/g), and q_e_ (mg/g) denote the contact time, mass of adsorbate normalized by mass of adsorbent at time t, and equilibrium, respectively. Experimental findings determined the following: the rate constants for the PFO and PSO, *k*_1_ (min^–1^) and *k*_2_ (g/(mg. min)), respectively; the IPD coefficient, kd (mg/(g.min^1/2^)); and Cd (mg/g) represents the constant associated with boundary layer thickness.

Various PFAS concentrations (1, 5, 10, 20, 50, 100, and 200 μg/L) for each of the ten PFAS were used in adsorption isotherm tests at 25 °C and 150 rpm for 8 h, with an adsorbent dose of 100 mg/L and a pH of 6.8. As seen in eqs (5)-([Disp-formula eq8]), four isotherm models—the Langmuir, Freundlich, Sips, and Toth—were employed to fit the adsorption data:^27,28^

5 $$\mathrm{Langmuir}:q_{e}=K_{L}q_{m}C_{e}/\left(1+K_{L}C_{e}\right)$$

6 $$\mathrm{Freundlich}:q_{e}=K_{F}C_{e}^{1/m}$$

7 $$\mathrm{Sips}:q_{e}=q_{m}{\left(K_{S}C_{e}\right)}^{1/n}/\left[1+{\left(K_{S}C_{e}\right)}^{1/n}\right]$$

8 $$\mathrm{Toth}:q_{e}={q_{m}\times K}_{T}\times C_{e}/{\left[1+{\left(K_{T}\times C_{e}\right)}^{t}\right]}^{1/t}$$

In this case, the quantity of adsorbate retained per gram of adsorbent is represented by q_e_ (mg/g), which stands for equilibrium uptake. In contrast, q_m_ (mg/g) defines the maximum adsorption capacity determined theoretically, and C_e_ (μg/L) denotes the adsorbate concentration at equilibrium. K_L_ (L/μg) represents the Langmuir constant (related to adsorption capacity), and K_F_ (mg·L^1/m^/(g·μg^1/m^)) represents the Freundlich constant (related to adsorption capacity and energy). Similarly, K_S_ (L/μg) is expressed as the adsorption affinity in the Sips model constant, and K_T_ (L/(g) certifies the Toth isotherm constant. In the Freundlich model, m represents surface heterogeneity, whereas in the Toth model, t characterizes the degree of system-level heterogeneity; the dimensionless index m indicates the favorability of adsorption.

Adsorption thermodynamic studies were conducted following the same procedure as above, except at two additional temperatures (35 and 45 °C). Thermodynamic parameters such as free energy change (ΔG), entropy change (ΔS), and enthalpy change (ΔH) were calculated according to eqs 9 and 10):^29^

9 $${△G}^{0}={△H}^{0}-T{△S}^{0}$$

10 $$\ln Kc=\frac{{-△G}^{0}}{RT}=\frac{{△S}^{0}}{R}-\frac{{△H}^{0}}{RT}$$

where △G^0^ = the standard Gibbs free energy change, usually expressed in kJ mol^–1^, △H^0^ = the standard enthalpy change, reported in J mol^–1^, △S^0^ = the standard entropy change, reported in J. mol^–1^ K^–1^, R = the universal gas constant, with a value of 8.314 J. mol^–1^ K^–1^, and T is the absolute temperature, given in Kelvin (K).

### 2.5 Selectivity Studies

We evaluated the impact of three environmental factors on the adsorption of PFAS. The variables consisted of pH levels ranging from 3 to 11, different ionic strengths with NaCl concentrations ranging from 1 to 200 mM, and different concentrations of natural organic matter (NOM) represented by humic acid from 1 to 100 mg/L. Each PFAS was tested at 10 μg/L with an experimental duration of 8 h. Furthermore, the adsorption behavior of PFAS in stormwater was investigated using a similar approach, except that each PFAS was intentionally spiked at a concentration of 0.05, 0.2, 0.5, 1, 5, and 10 μg/L. The adsorption test lasted for 1 h.

### 2.6 Characterization

A scanning electron microscope integrated with energy dispersive X-ray spectroscopy (SEM-EDS, Zeiss LEO 1550, Oberkochen, Germany; Bruker Quantax XFlash 6, Billerica, MA, USA) was used for the morphological and compositional examinations of the materials. Functional group analysis of the adsorbent samples before and after PFAS adsorption was carried out using Fourier transform infrared spectroscopy (FTIR; PerkinElmer Spectrum 100, Waltham, MA, USA). With a resolution of 1 cm^–1^, the spectral data were acquired within the 4,000–650 cm^–1^ spectral band. Nitrogen gas adsorption isotherms were obtained at a temperature of 77 K using a 3Flex gas adsorption analyzer (Micromeritics, Norcross, GA, USA). Before the N_2_ gas adsorption examination, the adsorbents underwent activation under decreased pressure and at a temperature of 50 °C (about 0.1 mbar) for 24 h. The Brunauer Emmett Teller (BET) technique was used to estimate the surface areas, while the pore size distributions were derived using density functional theory. A Water Contact Angle Goniometer made by Ossila Ltd. of Sheffield, UK, was used to measure the angle. The measurement involved the dispensing and withdrawal of around 2 μL of sessile water droplets using a syringe. A clean glass slide was used to gently flatten the epidermal layer using Kapton tape before it was conditioned at 20 °C to evaporate any remaining surface moisture. The XRD was fitted with a graphite monochromator and a D/teX Ultra one-dimensional silicon strip detector. The investigated crystalline samples were pulverized and positioned in zero-background holders, which were subjected to scanning with a 0.01° increment. The Malvern Zetasizer Nano-ZS analyzer (Malvern Panalytical Ltd., Malvern, UK) was used to measure the particle size distribution and ζ potential at a neutral pH and room temperature. X-ray Photoelectron Spectroscopy (XPS) spectra were collected with a PHI Quantera Hybrid system to analyze the chemical state of the elements present on the surface. The instrument is equipped with an Al K Alpha source (1486.6 eV). The diameter of the beam was 200 μm, and the data were collected using 26 eV pass energy at 45 deg takeoff angle. Dual charge compensation was used, and the XPS peak analysis was performed using Casa XPS software. The detailed chemical and PFAS analysis information is presented in Text S3 and Text S4.

### 2.7 Regeneration and Reuse of PVA-CTAC ENM

To assess the feasibility of regenerating and reusing the spent PVA-CTAC ENM, PFAS recovery experiments were conducted subsequent to the first adsorption testing. In each experiment, 5 mg of fresh PVA-CTAC ENM (equivalent to 100 mg/L in 50 mL solution) was subjected to a mixed solution of 10 PFAS for 8 h. Following adsorption, the PFAS-laden PVA-CTAC ENM was isolated by centrifugation and underwent a regeneration process. The adsorbent underwent treatment with 1% methanolic ammonium hydroxide in three successive extraction phases, each lasting 30 min, in compliance with EPA Method 1633.^30^ The 1% methanolic NH_4_OH can effectively desorb anionic PFAS by combining polarity-driven solubilization in methanol and ion-exchange with ammonium hydroxide. Following extraction, the ENM was meticulously washed with DI water, reconditioned in a 1% CTAC solution, and dried overnight at 40 °C for reuse. The tests on regeneration and reuse were conducted for three cycles, respectively. The mass of PFAS adsorbed or desorbed in each cycle was calculated from the total PFAS introduced into 50 mL of solution, using equations (S1)–(S4) shown in Text S5. All methanol extracts, wash solutions, and postadsorption samples were tested for PFAS using LC-MS/MS, in accordance with the protocol detailed in Text S4. The limits of detection (LODs) for each PFAS, along with other LC-MS/MS quantification parameters, are presented in Table S4.

## 3 Results and Discussion

Herein, the electrospinning procedure was employed to fabricate a novel PVA-CTAC nanofiber adsorptive membrane. As shown in Scheme 1, a stable and uniform spinning solution, including PVA polymer and CTAC, was blended homogeneously. The PVA has a negative charge and numerous OH– groups at every other carbon in its chain. In contrast, CTAC is a cationic surfactant including a quaternary ammonium headgroup with three methyl substituents and a lengthy hydrophobic tail of 16 carbon atoms. Following electrospinning, electrostatic and hydrogen-bonding interactions occurred between the hydroxyl groups of PVA and the quaternary ammonium groups of CTAC, enabling the surfactant molecules to be uniformly distributed and physically incorporated into the polymer matrix. Subsequent GA cross-linking formed acetal linkages among PVA chains, effectively entrapping CTAC within the nanofiber network and resulting in stable structural stability and positive surface charge for PFAS adsorption.^31^

**Scheme 1:**
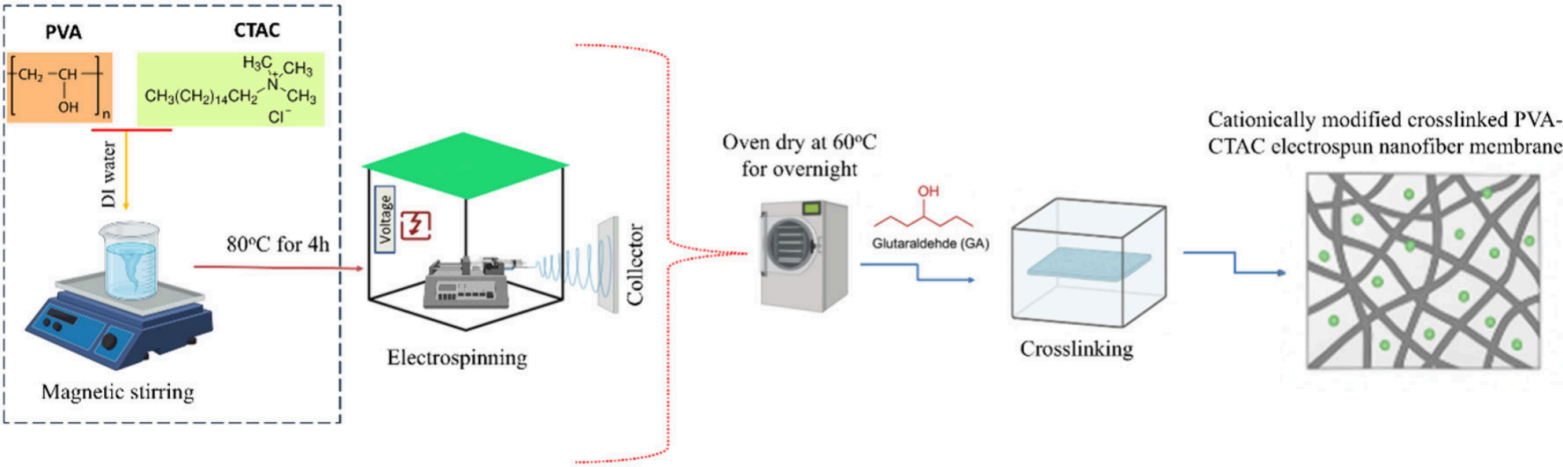
PVA-Based Electrospun Nanofiber Membrane Synthesis Approach

### 3.1 Selection of Adsorbents

The use of PVA in this investigation was motivated by its biocompatibility, cost-effectiveness, and the ability to form a structured assembly of electrospun nanofibers. The evaluation and enhancement of PVA-based electrospun nanofibrous membranes started with an extensive examination of components and solution modifications potentially applicable to the adsorption of PFAS from aqueous environments. In Figure 1a, the pristine PVA nanofiber membrane shows negligible adsorption for short to medium chain PFAS, while considerable removal efficiency is observed for long-chain PFAS such as PFDA (∼ 100% within 1h), PFNA (around 70%), PFOS (more than 80% after 8h), and only 20% observed for PFOA. Despite the highly negatively charged surface of PVA ENM,^32^ the increased effectiveness in removing long-chain PFAS from the solution implies that hydrophobic interactions played a significant role.

**Figure 1:**
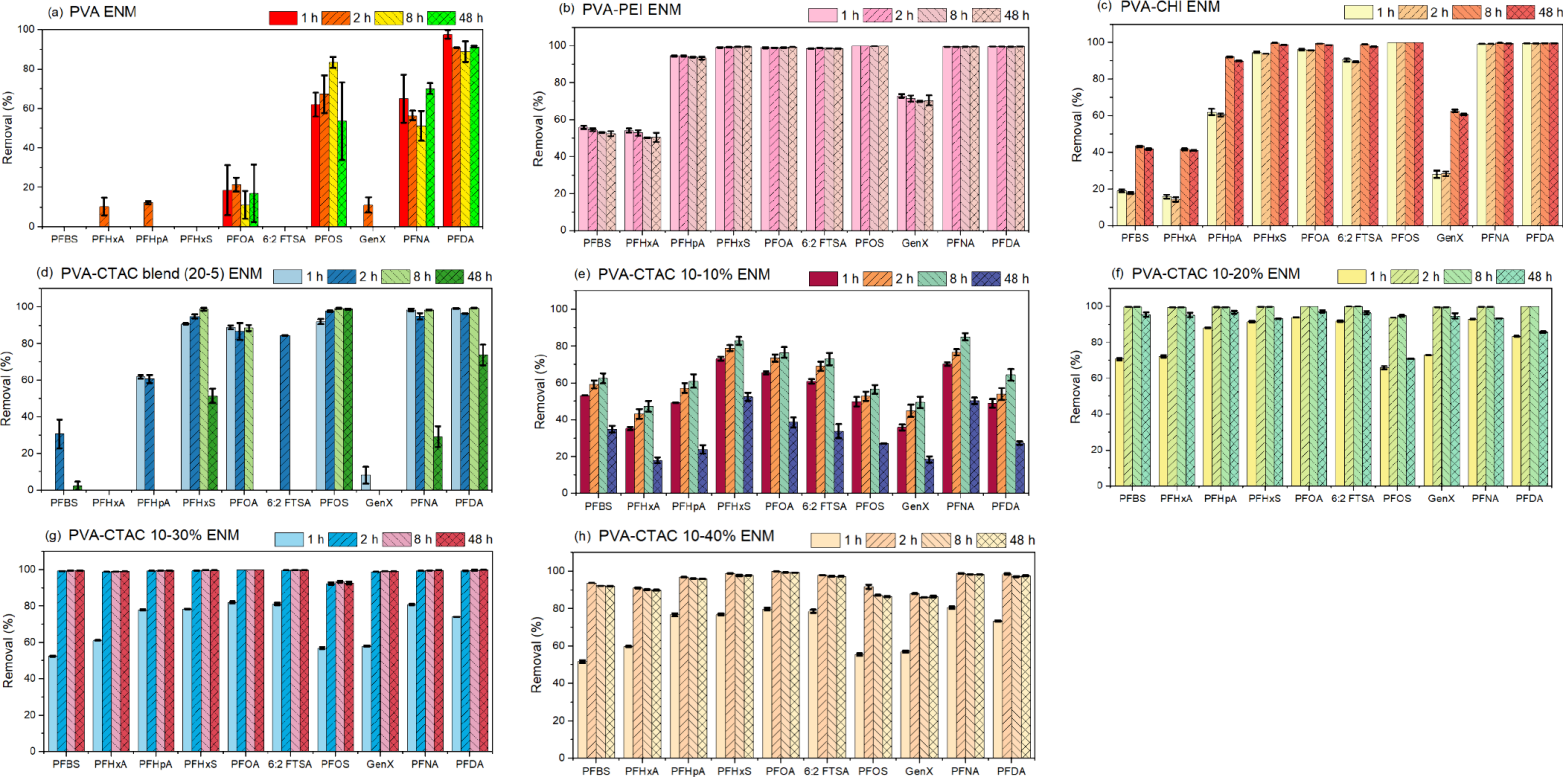
PFAS adsorption performance at 1, 2, 8, and 48 h. (a) PVA ENM; (b) PVA-PEI ENM; (c) PVA-CHI ENM; (d) PVA-CTAC blend (20–5) ENM; (e) PVA-CTAC 10–10% ENM; (f) PVA-CTAC 10–20% ENM; (g) PVA-CTAC 10–30% ENM; and (h) PVA-CTAC 10–40% ENM. Experimental conditions: initial PFAS concentration of 10 μg/L and adsorbent dose of 100 mg/L. Error bars denote the standard deviation of three independent replicates.

Recent developments have shown that cationic surfaces are preferable for capturing anionic PFAS; herein, we employed chitosan, a positively charged biopolymer, to enhance the performance of the PVA-chitosan blended ENM toward PFAS removal. It is well-established that PVA strongly interacts with chitosan through hydrogen bonding,^33^ which facilitates the adsorption performance as shown in Figure 1b. In terms of long-chain PFAS removal, PVA-CHI ENM demonstrated nearly 100% for PFHxS, PFOA, PFOS, PFNA, PFDA, and around 90% for PFHpA after 8h, which is in agreement with previous studies reporting similarly positive effects of chitosan for enhancing removal of long-chain PFAS from water metrics.^34^ However, the removal of short-chain PFAS is below 60%, possibly due to a lack of adsorption binding sites. On the other hand, as shown in Figure 1c, the incorporation of polyethylenimine (PEI), a branched polycationic polymer with PVA, led to more than 50% removal of PFBS and PFHxA, and 70% for GenX.

It has been demonstrated that cationic surfactants efficiently regulate the physicochemical properties of a given adsorbent, leading to high removal of PFAS. Initially, the volume-to-volume ratio of PVA-CTAC at 20:5 resulted in the ENM that had a higher removal of PFAS than the pristine PVA ENM (Figure 1d). The removal efficiency for PFNA and PFDA was observed to be nearly 100% within 1h and close to 100% for PFHxS and PFOS after 8h. To improve the removal efficiency of PFBS, PFHpA, and 6:2 FTSA, we decided to use wt % for the preparation of PVA-CTAC ENM. Among the four weight-based ratios, PVA-CTAC at 10–10, 10–20, 10–30, and 10–40, the 10–10 wt % (Figure 1e) had a considerable improvement for short and long-chain PFAS, and 10–20% (Figure 1f) and 10–40% PVA-CTAC ENM (Figure 1h) removed all PFAS nearly 90–100%, but desorption was observed for some PFAS after 8 h. The desorption is likely due to partial saturation of adsorption sites or weakly bound molecules. Interestingly, the 10–30% PVA-CTAC ENM (Figure 1g) showed almost 100% removal for all PFAS tested, except PFOS at 95%. Given the strong adsorption of all target PFAS without desorption, the 10–30% PVA-CTAC ENM was further investigated, as discussed in the following section.

### 3.2 Characterization of Adsorbents

The surface morphology and elemental composition of the as-prepared ENMs were analyzed using SEM and EDS. Figure 2a and 2d present a typical SEM image of the pristine PVA membrane, which shows long fibers with a diameter of 463 nm that are randomly oriented without any bead formation. On the other hand, the PVA-CTAC ENM in Figure 2b and 2e had a decreased diameter (184.7 nm) compared to the pristine PVA ENM and featured an interweaving network and overlapping structure, which could strongly provide a plethora of porous and hollow areas that were crucial for the adsorption of PFAS.^35,36^ The reduction in diameter of the PVA-CTAC ENM is consistent with the known effects of surfactant doping, which increase solution conductivity and reduce surface tension, thereby producing finer fibers during electrospinning.^37–39^ After adsorbing PFAS species, there was a formation of a shell-like cover around the surface of the fibers (Figure 2c and 2f). Prior research has shown that nanofiber-based adsorbents have significantly superior adsorption capabilities compared to materials with similar compositions but differing morphologies. For instance, α-MnO_2_ nanofibers exhibited an As­(V) adsorption capacity of 60.19 mg/g, but a bulk MnO_2_–organic bentonite composite had a far lower capacity of just 3.1 mg/g, indicating a 94.8% decrease. The EDS examination of the pristine PVA ENM (Figure S1a,d) identified two primary constituents: carbon (C) and oxygen (O), comprising 64.84% and 27.01% of the atomic composition, respectively. The data indicate that the electrospinning method maintained the purity of the PVA solution, free from environmental pollutants.^40^ As depicted in Figure S1b,c, the weight percent of 12.92% for Cl and 15.62% for N confirmed the participation of CTAC onto the PVA chain^41^ in the synthesis of the membrane. After that, the PFAS adsorption through the membrane was confirmed by the presence of F (0.31%), as shown in Figure S1c,f.^42^ The XRD results (Figure S2a) showed a typical peak relevant to the (101) plane of PVA corresponding to the amorphous^43^ and semicrystalline nature of PVA.^44^ Additionally, the PVA-CTAC ENM (Figure S2b) consisted of two diffraction peaks at 2θ = 9° and 12°, demonstrating that the inclusion of CTAC relatively influenced the crystallinity of pristine PVA.^23^ It can be said that, since the peak diffraction of 20° is still present in the PVA-CTAC, PVA could maintain its partially crystalline nature while accommodating new crystalline regions due to the introduction of CTAC. Although the degree of GA cross-linking was not quantitatively measured in this study, FTIR confirmed the formation of characteristic cross-linking bonds, and no abnormal adsorption behavior attributable to residual GA was observed. Quantifying cross-linking density and residual GA will be further evaluated in future work to strengthen assessments of membrane stability and surface chemistry.

**Figure 2:**
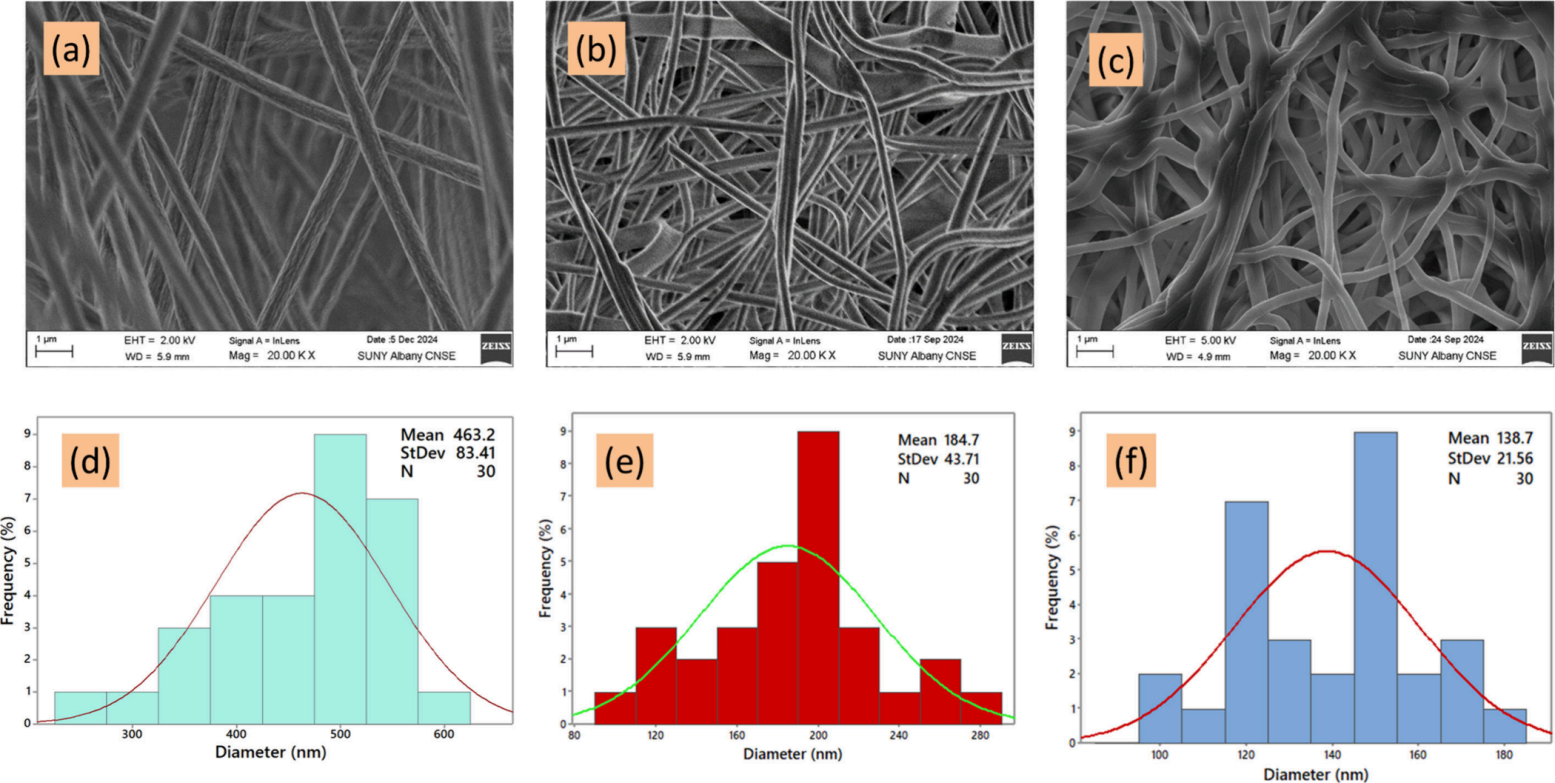
SEM and diameter distribution of ENM, (a, d) pristine PVA ENM, (b, e) PVA-CTAC ENM before adsorption, and (c, f) after adsorption.

Table S5 summarizes the results of the water contact angle and BET studies. Surface area (1.65 ± 0.12 m^2^/g) and pore volume (0.3807 cm^3^/g) of pristine PVA ENM were found to be rather low, in line with its thick polymeric structure. The surface area and total pore volume both rose significantly to 5.50 ± 0.03 m^2^/g and 0.9607 cm^3^/g, respectively, after CTAC was added. The increase in surface area for PVA-CTAC ENM reflects the more porous bulk nanofiber network, including interfiber porosity, and is consistent with the smaller fiber diameter and enhanced porosity from surfactant doping.^45^

Utilizing GA cross-linking allowed the nanofibers to be more stabilized and their wettability to be customized. A larger contact angle showed that cross-linked PVA-CTAC ENM was more hydrophobic than its non-cross-linked equivalent. The acetal bond synthesis with GA consumes hydroxyl groups, thereby decreasing surface polarity and inhibiting excessive water absorption, which explains this phenomenon.^46^ Incorporating CTAC and cross-linking with GA allows PVA-based nanofibrous membranes to have surface chemistry and wettability tuned.

### 3.3 Adsorption Kinetics, Isotherms, and Thermodynamic Studies

The results from the batch adsorption kinetic experiments of PFAS using PVA-CTAC ENM are outlined in Figure 3a. Interestingly, there was a notable amount of adsorption of total PFAS within the first 60 s (as shown in Figure 3a, insert), and the adsorption equilibrium was achieved within 12 min. The PFO, PSO, and IPD models were used to accurately match the kinetic data and better understand the adsorption kinetics. The linear forms of the models were used to evaluate the degree of correlation in the fitting. The linearized PFO model yielded R^2^ values ranging from 0.144 to 0.612, whereas the IPD model produced R^2^ values ranging from 0.321 to 0.408 (Figures S3 and S4). The linearized PSO model, on the other hand, showed R^2^ values ranging from 0.999 to 1 for each of the 10 PFAS (Figure S5). The PSO model provides a good explanation for the adsorption kinetics of the studied chemicals, as evidenced by this near-perfect correlation. Similarly, Sun et al.^47^ found similar patterns in kinetics and showed that the PSO model offered the best description of capturing PFAS mixtures using TPU/PEI electrospun nanofiber membranes. It is important to clarify that although the PSO model is often associated with chemisorption, a good fit to this model does not necessarily imply true chemical bonding or electron sharing/transfer. Indeed, several studies have shown that PSO kinetics can also arise from physical processes such as diffusion-limited adsorption.^48,49^

**Figure 3:**
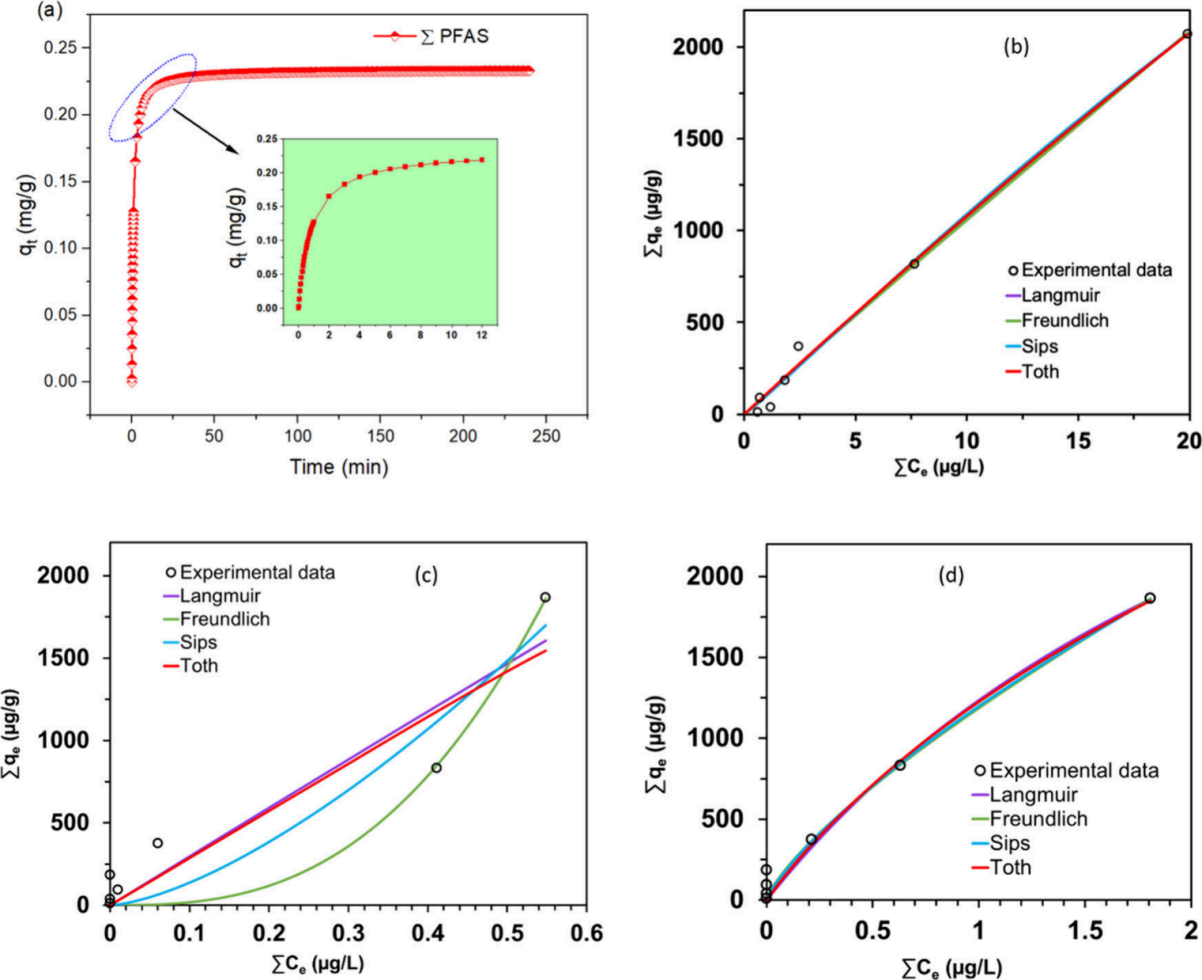
(a) Kinetic profile of total PFAS absorption by PVA-CTAC ENM at an initial concentration of 10 μg/L. Colored markers represent experimental results at each sampling interval, while the fitted PSO model is shown as solid curves. Equilibrium adsorption isotherms of total PFAS on GNP-CTAC were investigated at (b) 25 °C, (c) 35 °C, and (d) 45 °C. Each data point represents the mean of three independent measurements.

Equilibrium isotherms are used to determine the maximum adsorption capacity of a material and to investigate the interaction mechanisms between adsorbate and adsorbent. Generally, equilibrium isotherms are fitted using well-known mathematical models, such as the Langmuir, Freundlich, Sips, and Toth models, in order to characterize the adsorption process. The Langmuir model posits monolayer adsorption at dynamic equilibrium, while the Freundlich model considers adsorption on heterogeneous surfaces. The Sips and Toth equations integrate aspects of both models, rendering them appropriate for systems characterized by surface heterogeneity.^50^ Adsorption equilibrium experiments were conducted at 25, 35, and 45 °C to assess the impact of temperature on PFAS uptake by the PVA–CTAC ENM. The equilibrium data (∑qe versus ∑Ce) were analyzed using various isotherm models, and the resulting parameters are detailed in Tables S6–S8. Model-predicted q_e_ values for each temperature are shown in Tables S9–S11, with the associated fits displayed in Figure 3b–d.

At 25 °C, the Langmuir model captured the trend toward saturation at higher concentrations, whereas the Freundlich model described the low-concentration region more accurately, reflecting surface heterogeneity. The Sips and Toth models provided excellent fits across the entire range, indicating that adsorption occurs on surfaces containing multiple classes of energetically diverse binding sites. When the temperature increased to 35 °C, adsorption improved substantially. The isotherm curvature became steeper, and both the Sips and Toth affinity constants increased sharply, consistent with the endothermic nature of PFAS adsorption. This temperature activates additional hydrophobic and fluorophilic interactions and enhances access to higher-energy binding domains. At this temperature, the Langmuir adsorption capacity (qm = 104.9 mg/g) was the highest among the three conditions, indicating that PFAS uptake is most favorable at moderately elevated temperatures. At 45 °C, adsorption remained efficient, but the pattern of the model fits changed. All four models converged closely, suggesting that thermal rearrangement of CTAC headgroups and alkyl domains produces a more uniform energetic environment. The Langmuir model yielded a much lower capacity (rounded to 5 mg/g). This decrease does not indicate weaker binding—in fact, the affinity constants (KL, KS, KT) increased—but rather reflects a reduction in multilayer or secondary adsorption contributions at this higher temperature, where increased thermal motion and CTAC reorganization limit the formation of PFAS–PFAS packing structures.

Rather than emphasizing minor numerical differences in R^2^ values, these results are interpreted in terms of adsorption mechanisms. The strong agreement between the Sips and Toth models across all temperatures confirms that PFAS interact with multiple site types on the PVA–CTAC ENM. High-affinity adsorption arises from electrostatic attraction between anionic PFAS headgroups and positively charged CTAC quaternary ammonium groups, combined with hydrophobic and fluorophilic interactions between PFAS tails and CTAC alkyl chains. Lower-affinity interactions originate from residual PVA hydroxyl regions. The observed transition from Freundlich-like behavior at low coverage to Langmuir-like behavior at higher coverage indicates that PFAS initially occupy the energetically strongest domains before progressively filling secondary sites.

Thermodynamic parameters derived from temperature-dependent equilibrium data (Table S12) corroborate these observations. The Gibbs free energy remained negative at all temperatures: −8.82, −15.38, and −21.94 kJ/mol at 298, 308, and 318 K, respectively—indicating that PFAS adsorption is spontaneous throughout the examined range. The positive enthalpy change (ΔH° = +186.67 kJ/·mol) confirms that adsorption is endothermic, and the positive entropy value (ΔS° = +0.656 kJ/·mol/·K) indicates substantial disordering at the solid–solution interface. Overall, the thermodynamic parameters corroborate the mechanistic interpretation derived from the isotherm analysis: PFAS adsorption on the PVA–CTAC ENM is spontaneous, endothermic, and predominantly entropy-driven, governed by a combination of electrostatic attraction and hydrophobic/fluorophilic interactions. These results provide a unified energetic framework that supports the proposed adsorption mechanism and reinforces the ability of CTAC-modified nanofibrous membranes to efficiently remove diverse PFAS under environmentally relevant conditions, compared with reported commercial adsorbents (Table S13).

### 3.4 Selectivity

The adsorption of PFAS onto adsorbents is highly pH dependent. As shown in Figure 4a, the PVA-CTAC ENM exhibited consistently high PFAS removal efficiencies across a wide pH range of 3–11 for most tested PFAS, including PFOA, PFHpA, PFHxS, and 6:2 FTSA, indicating that the adsorption performance was largely unaffected by pH variations. This stability suggests that the adsorption is primarily governed by strong hydrophobic interactions between the fluorinated chains of PFAS and the hydrophobic domains of the membrane.^51^ For PFBS, PFHxA, and GenX, the decline in removal at elevated pH levels may be attributed to increased electrostatic repulsion resulting from the higher concentration of hydroxyl ions, which enhances ionic competition and thus reduces removal efficiency. Notably, when the pH was 3, the removal efficiencies of long-chain PFAS, C8–C9 PFOS, PFNA, and PFDA were reduced significantly compared to neutral or alkaline conditions. Given the wide range of p*K*_a_ values reported for each PFAS, for example, the p*K*_a_ of PFOS is between −3.4 and 1.2,^52^ The observed decrease in adsorption cannot be explained solely by protonation effects, and possible membrane deterioration under strongly acidic conditions cannot be excluded. However, this postulation does not explain the unaffected adsorption of short-chain PFAS. Although such low pH conditions are rarely encountered in the environment (typically pH 5.5–8.5), the adsorption decrease of these three PFAS warrants further investigation, such as through FTIR and SEM analyses. As illustrated in Figure 4b, the zeta potential of the PVA-CTAC ENM decreased with increasing pH and exhibited a point of zero charge (PZC) at pH 10.02, indicating that the membrane surface should carry a positive charge under typical environmental pH of 5.5–8.5. This positively charged surface could potentially facilitate strong electrostatic interactions with anionic PFAS, thereby enhancing adsorption efficiency for all tested PFAS species.

**Figure 4:**
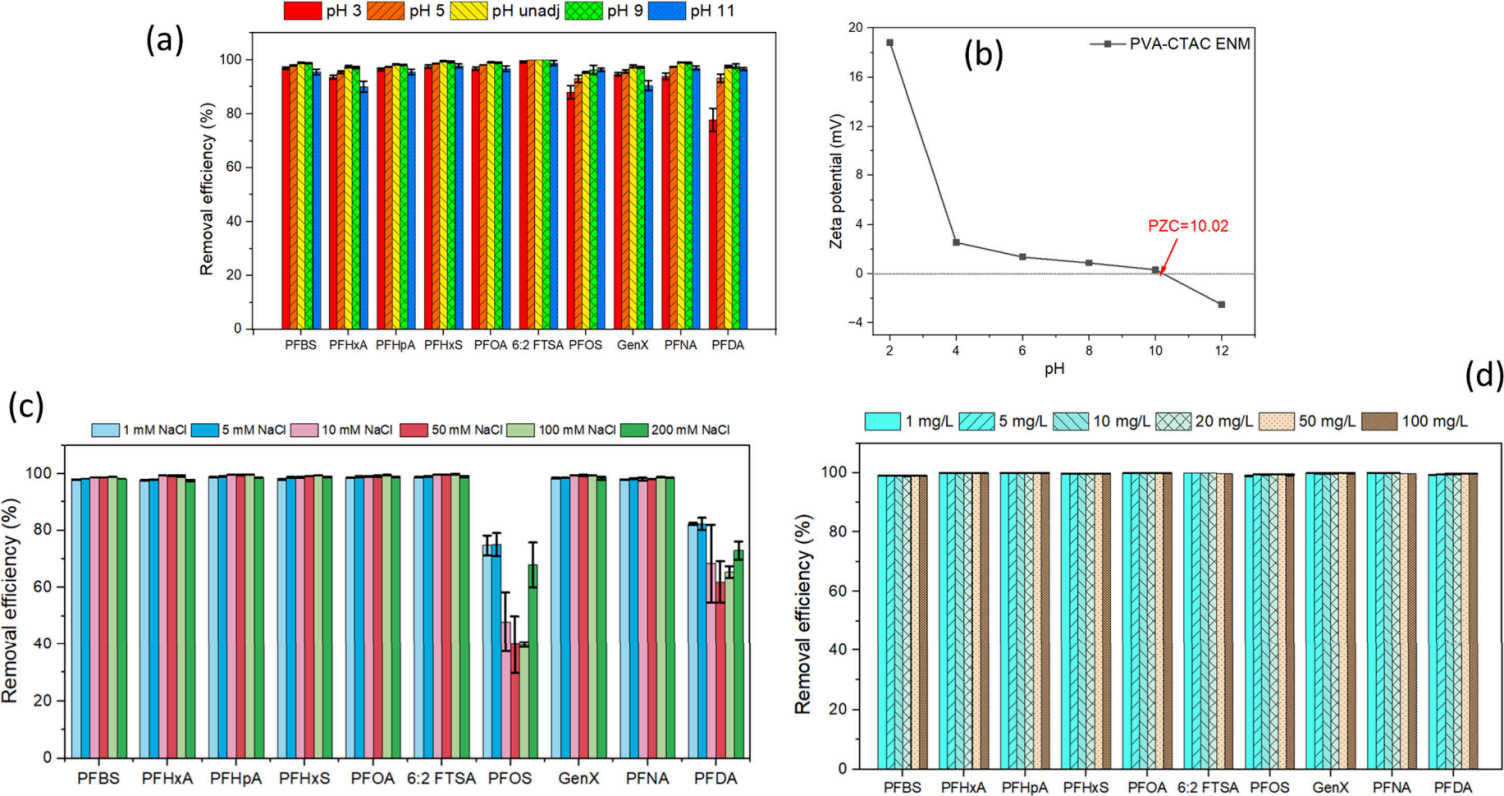
Effect of (a) pH, (b) zeta potential, (c) NaCl, and (d) NOM, PFAS adsorption by PVA-CTAC ENM.

The effect of ionic strength on PFAS removal was also investigated by varying the NaCl concentration from 1 to 200 mM (Figure 4c). The membrane demonstrated above 95% removal of PFBS, PFHxA, PFHpA, PFHxS, GenX, PFNA, 6:2 FTSA, and PFOA across a wide range of ionic strengths (1 to 200 mM NaCl). However, PFOS and PFDA exhibited a notable decrease in removal efficiency at higher salt concentrations, particularly beyond 10 mM NaCl, suggesting that ionic strength plays a more pronounced role for longer-chain PFAS. Several competitive ion processes may contribute to this trend. First, increasing the Na^+^ concentration partially reduces the positive charge on the membrane’s quaternary ammonium groups, weakening electrostatic attraction between the membrane and the anionic headgroups of PFOS and PFDA. Second, Cl^–^ ions compete with PFAS for access to cationic binding domains, which can shift adsorption toward weaker secondary sites. In addition, high ionic strength compresses the electrical double layer at the membrane surface, reducing the effective interaction distance for long-chain PFAS whose hydrophobic tails rely on partial alignment or ordering at the interface. Because PFOS and PFDA have larger hydrophobic regions and stronger interfacial organization than short-chain PFAS, they are more sensitive to these ion-dependent changes. Together, these phenomena clearly explained their decreased removal efficiency under high-salinity conditions, while shorter-chain PFAS, whose adsorption relies more on hydrophobic and site-specific interactions, remain less affected. These results demonstrate that PVA-CTAC ENM is a robust and versatile adsorbent capable of efficiently capturing PFAS across environmentally relevant water matrices, including surface water (ionic strength ≈1–5 mM), groundwater (≈1–20 mM), and seawater (≈700 mM).^53^

As shown in Figure 4d, the PVA–CTAC ENM maintained high PFAS removal (>90–100%) across NOM concentrations ranging from 1 to 100 mg/L. This stability is likely due to the strong electrostatic interaction between anionic PFAS headgroups and the quaternary ammonium sites, which is not easily displaced by bulky, weakly charged NOM molecules. The hydrophobic C16 tail of CTAC further strengthens PFAS–membrane interactions through combined fluorophilic and hydrophobic association, while NOM, which contains mainly aromatic and oxygen-containing functional groups, exhibits weaker affinity for these hydrophobic domains. Additionally, the nanofibrous architecture provides primarily external and easily accessible adsorption sites, reducing the opportunity for NOM accumulation and fouling. Together, these characteristics explain the minimal impact of NOM on PFAS removal by the PVA–CTAC ENM. Moreover, it is known that surface water and wastewater contain a substantial quantity of NOM, which could interfere with the removal of PFAS during adsorption.^54^

Evaluation of the adsorption kinetics and NOM tolerance indicates that mass-transfer limitations are not the primary constraint for PFAS uptake by the PVA–CTAC ENM. The rapid initial removal and excellent agreement with the pseudo-second-order model suggest that adsorption is governed mainly by surface interactions rather than slow intrafiber diffusion. In addition, the membrane retained >90–100% removal across NOM concentrations up to 100 mg L^–1^, demonstrating that competitive displacement by organic matter is minimal. The strong electrostatic attraction between anionic PFAS headgroups and the quaternary ammonium sites, together with hydrophobic association with the CTAC tail, dominates the adsorption process and reduces the influence of NOM competition. These observations suggest that surface-controlled mechanisms prevail over both mass-transfer limitations and organic matter interference under the tested conditions.

### 3.5 PFAS Removal from Stormwater at Trace Concentrations

To more precisely assess the efficacy of PVA-CTAC ENM under actual settings, adsorption studies were conducted using PFAS-contaminated rainwater. The stormwater exhibited a concentration of PFAS ranging from 4 to 24 ng/L (Table S3). The adsorption efficiency of PVA-CTAC ENM was relatively low for both short- and long-chain PFAS at an initial concentration of 50 ng/L. As the concentration rose to 5–10 μg/L, there was a significant enhancement in removal performance, with overall removal efficiencies falling between 80% and 90% for all compounds (Figure 5). The impact of carbon chain length on adsorption was clear, with longer-chain PFAS typically demonstrating greater removal efficiency than their short-chain counterparts. PFOS and PFDA showed lower removal efficiencies, likely due to a limited number of available binding sites on the membrane for these specific species. This implies that the chemicals in the stormwater negatively affected membrane’s effectiveness in removing PFAS. According to Table S3, the collected stormwater had a fluoride concentration of 6.66 × 10^–3^ mg/L and a phosphate concentration of 2.37 × 10^–2^ mg/L. The total nitrogen (TN) content measured was 8.5 mg/L, which is above the normal levels documented for precipitation and stormwater.^55,56^ The total organic carbon (TOC) level was much higher than the worldwide average TOC concentration in lake water (5.578 mg/L).^57^ The presence of both anions and organic matter competes with PFAS for adsorption sites, hence substantially reducing the material’s adsorption effectiveness.^58,59^ In contrast to previous studies that have shown a lower ability of other adsorbents to remove PFAS from natural water,^60,61^ the PVA-CTAC ENM showed significant promise in effectively capturing PFAS in real-world water conditions. When comparing the adsorption in both DI water and stormwater at identical spiked concentrations (10 μg/L) and an adsorbent dose of 100 mg/L (Figures 1g and 5), the ENM consistently achieved ∼90% removal for all PFAS, except PFOS and PFDA. This performance indicates only moderate matrix interference from coexisting ions and organic matter and highlights PVA-CTAC ENM as a superior candidate for effectively eliminating PFAS in actual water.

**Figure 5:**
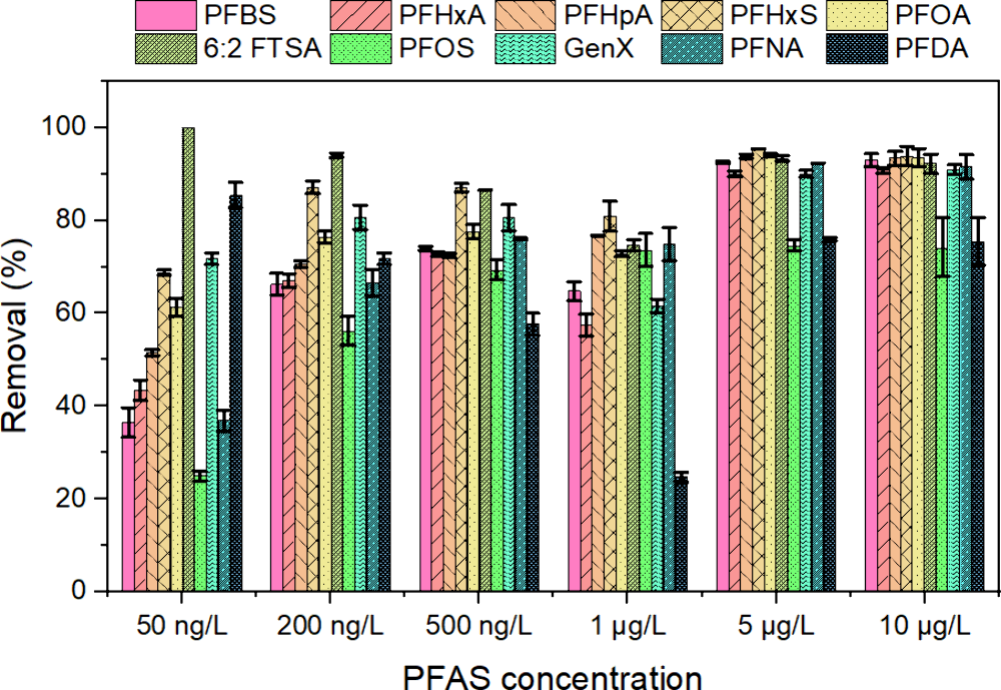
Removal of PFAS from stormwater by PVA-CTAC ENM. Each PFAS was spiked at concentrations of 50, 200, and 500 ng/L, as well as 1, 5, and 10 μg/L. Removal efficiencies were calculated from the measured concentrations, which showed slight variation from the spiked values. The adsorbent dosage was 100 mg/L, and the pH of the stormwater was left unadjusted. Error bars show the standard deviation of three independent replicates.

### 3.6 Regeneration and Reusability

The adsorbent’s regeneration capacity was evaluated during three successive adsorption–desorption cycles for a range of PFAS, as seen in Figure 6 and Table S14. The material demonstrated significant reusability, sustaining elevated removal efficiencies (R%) for the majority of chemicals across the cycles. PFBS, PFHxA, PFHpA, PFOA, 6:2 FTS, GenX, PFNA, and PFDA exhibited little variation, with recovery consistently at or above 95%. A decrease in removal effectiveness for PFHxS was observed during Cycles 2 and 3, possibly due to partial pore blockage occurring after repeated adsorption–desorption cycles. PFOS was originally shown to have marginally reduced recovery in Cycle 1, but thereafter demonstrated enhanced performance in subsequent cycles, indicating that initially inaccessible sites became available after the first regeneration. Although postregeneration FTIR or XPS was not performed, such characterization could confirm this mechanism, and future studies will include these analyses to verify structural or chemical changes upon reuse. Overall, the findings collectively indicate that the PVA-CTAC ENM can be efficiently regenerated and reused with no loss of efficiency, highlighting its potential for sustainable removal of short- and long-chain PFAS in real water.

**Figure 6:**
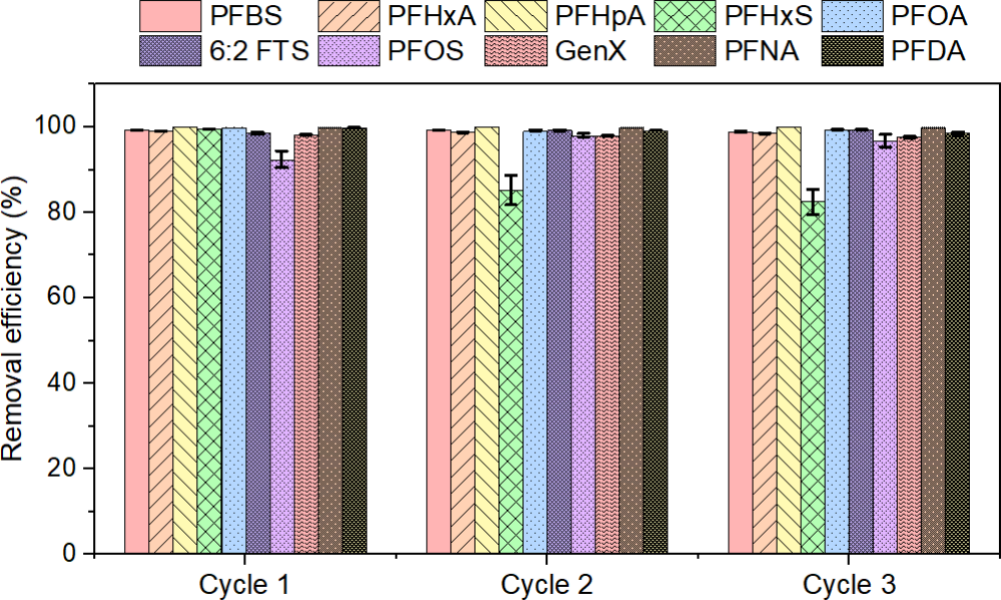
Percentage elimination of PFAS combinations during three regeneration–reuse cycles. Test parameters: starting concentration of PFAS, 10 μg/L; dosage of adsorbent, 100 mg/L; volume of solution, 50 mL; contact time, 8 h; and shaking at 150 rpm at room temperature. A three-tailed test with standard deviations shown by error bars.

### 3.7 Insights into Adsorption Mechanism

To further investigate the adsorption mechanisms, the FTIR and XPS measurements were employed. Based on the FTIR analysis, various functional groups were detected on the PVA-CTAC ENM. Figure 7 illustrates that for pristine PVA, a wide band at 3296 cm^–1^ (region I) corresponds to O–H stretching vibrations, while the band at 1647 cm^–1^ (region III) is ascribed to O–H bending.^62^ The absorption at 2942 cm^–1^ (region II) is due to the asymmetric and symmetric stretching of −CH_2_– groups, the band at 1425 cm^–1^ (region IV) pertains to −CH_2_– bending, and the peak at 1092 cm^–1^ (region V) relates to C–O stretching vibrations.^63^ In PVA-CTAC ENM, the O–H stretching peak changed to 3371 cm^–1^, demonstrating a distinct blue shift compared to pure PVA, signifying modified hydrogen-bonding interactions. This transition indicates that hydrogen bonding between hydroxyl groups in PVA chains was diminished during chemical cross-linking with GA. In the cross-linked PVA-CTAC ENM, the −CH_2_– stretching bands are divided into peaks at 2922 and 2852 cm^–1^ (region II), exhibiting markedly increased intensities attributed to the incorporation of −O–CH_2_–O– groups by acetalization.^64^ In addition, the peak at 1322 cm^–1^ is attributed to C–N functional groups, and 720 cm^–1^ implies the existence of a CTAC chain in the PVA-CTAC ENM.^65^ Importantly, an emergence of a 1241 cm^–1^ band corresponding to C–F bonds of PFAS^66^ indicated successful adsorption of PFAS onto the membrane surface.

**Figure 7:**
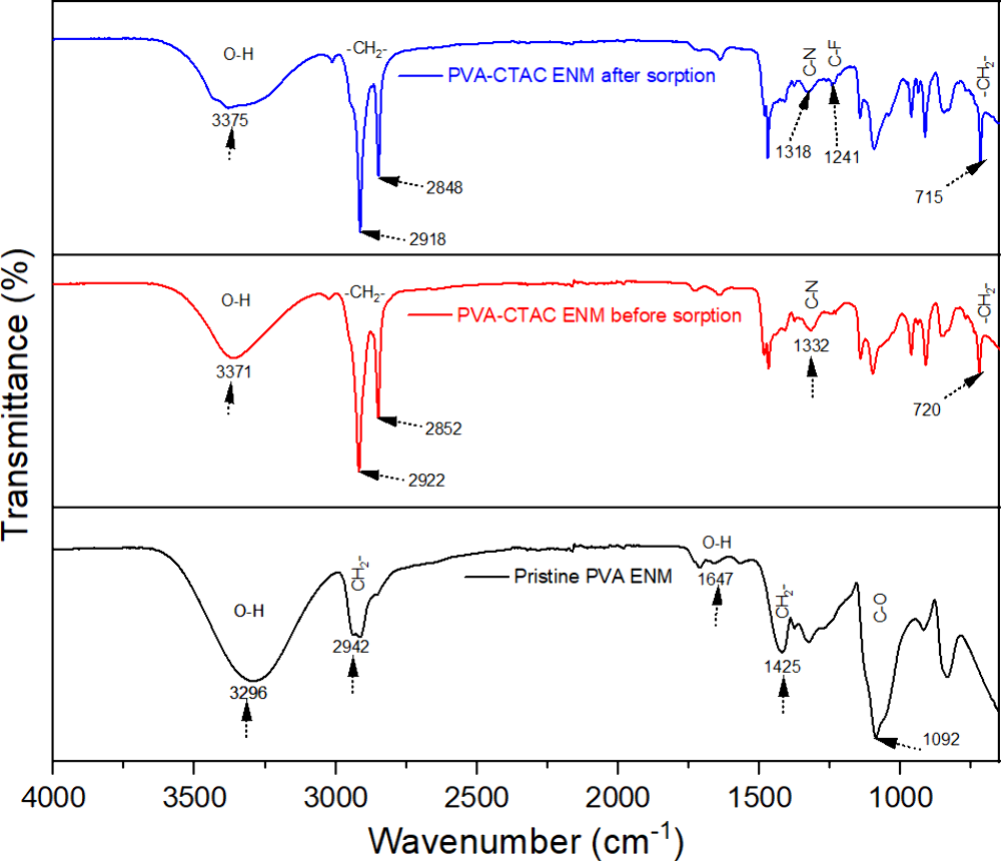
FTIR spectrum of pristine ENM and PVA-CTAC before and after adsorption.

Figure S6 shows the XPS survey scans of the PVA-CTAC ENM before and after PFAS adsorption. The atomic concentrations of the elements are summarized in Table S15. The pristine material was primarily composed of carbon (87.8%), derived from the lengthy backbone chains of PVA and CTAC. Additionally, an oxygen signal was detected from the PVA hydroxyl groups, whereas the nitrogen and chlorine peaks came from the head of the CTAC component, verifying its successful incorporation. After exposing PVA-CTAC to PFAS, a strong presence of fluorine was detected at an atomic concentration of 8.6%, highlighting the membrane’s effectiveness in absorbing PFAS.

High-resolution XPS spectra of the PVA-CTAC ENM before and after PFAS adsorption are presented in Figures 8 and 9, respectively. All the regions were calibrated based on the C 1s main peak (C–C/C–H) at 285.0 eV, given the material’s polymeric structure. Besides the C–C/C-H peak, the C 1s region of the pristine membrane showed a second peak at 286.23 eV attributed to C–N bonds in the quaternary ammonium group of CTAC and to C–OH bonds in PVA. The two bonds were expected to appear at similar binding energies; therefore, further deconvolution was not possible. A new peak at 292.15 eV in the C 1s spectrum after adsorption corresponded to C–F bonds. This suggested the successful capture of PFAS while the PVA-CTAC membrane retained its integrity.

**Figure 8:**
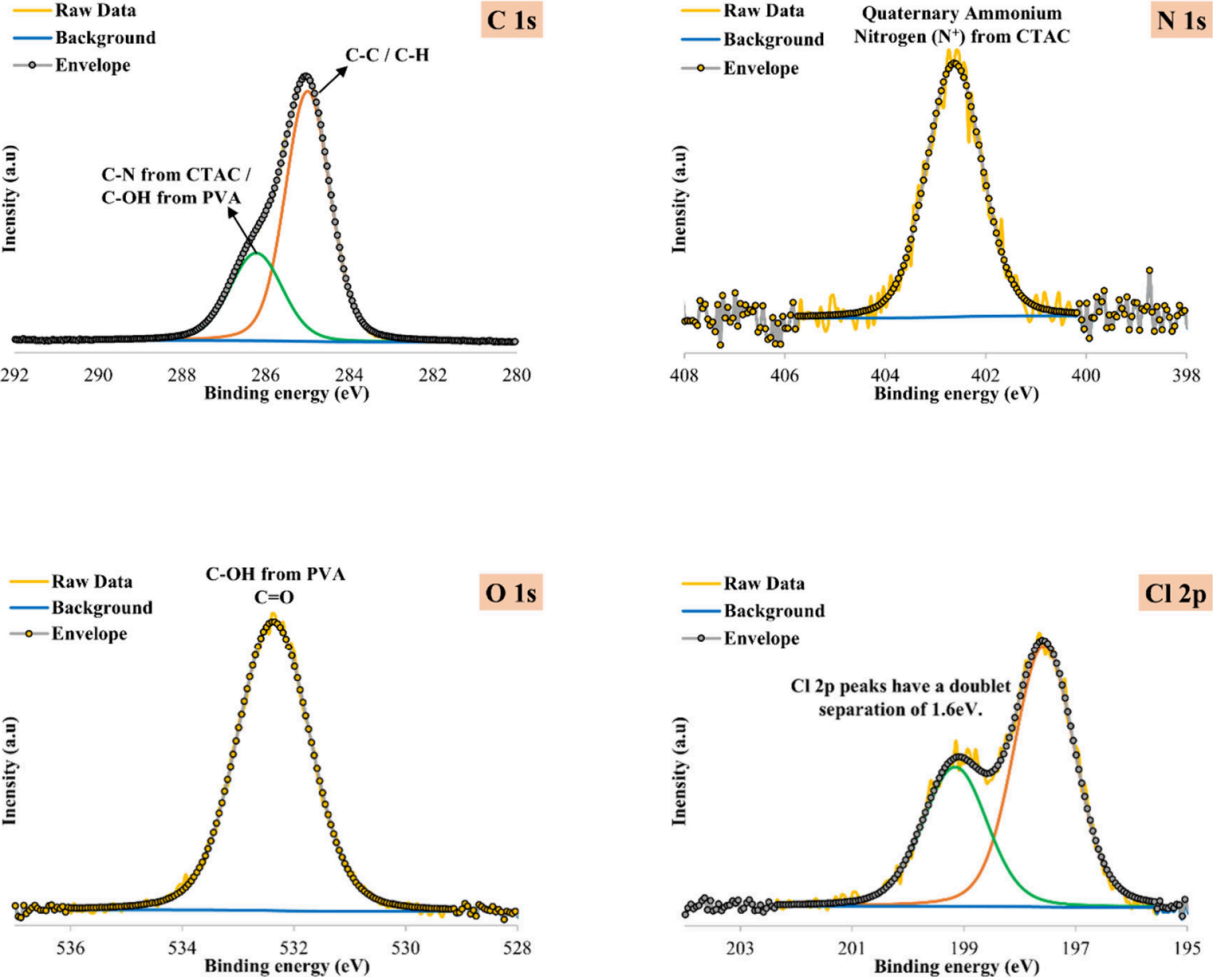
C 1s, O 1s, N 1s, and Cl 2p spectra of PVA-CTAC ENM (before adsorption).

**Figure 9:**
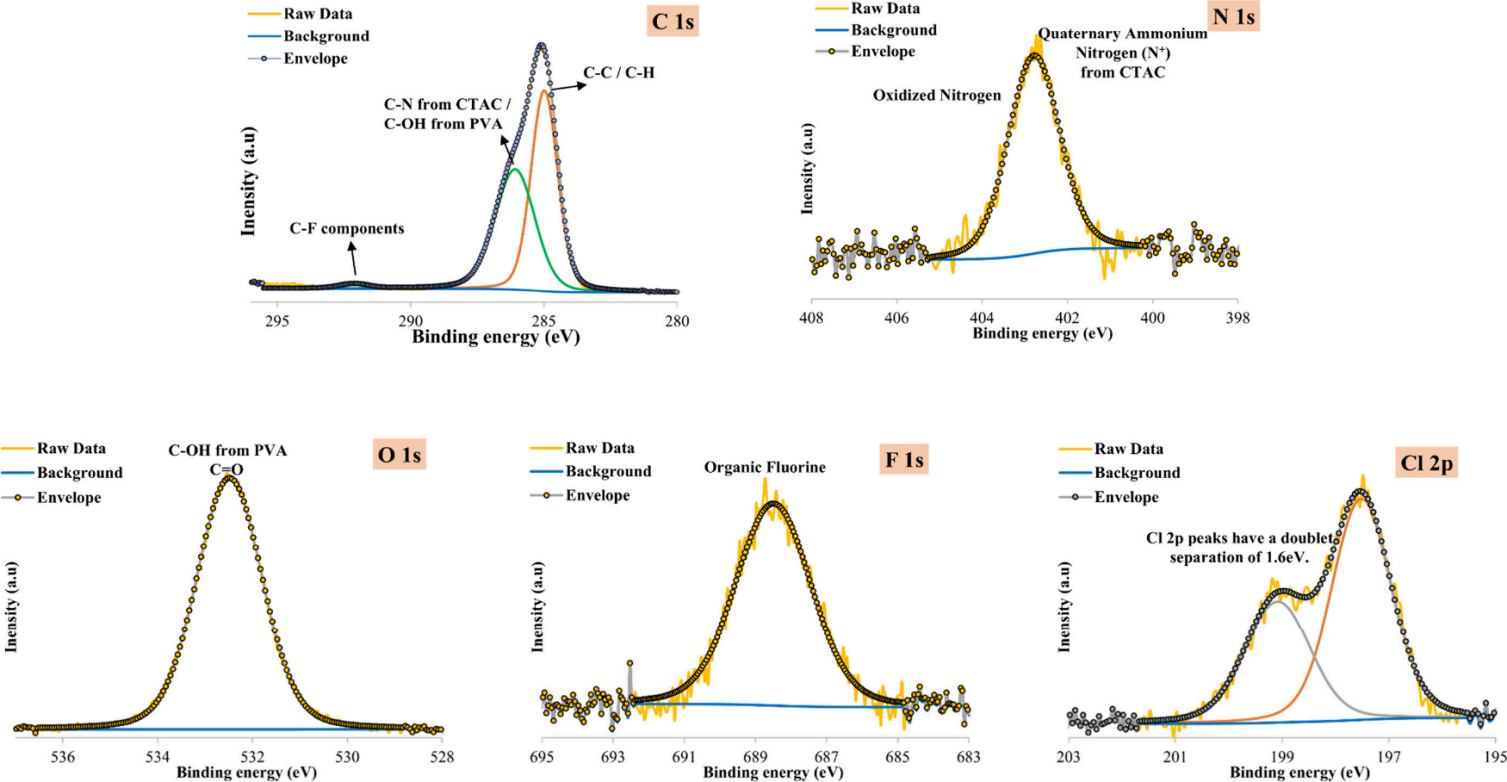
C 1s, O 1s, N 1s, F 1s, and Cl 2p spectra of PVA-CTAC ENM (after adsorption).

The incorporation of CTAC in the PVA structure was also confirmed by the presence of a peak at 402.63 eV in the N 1s spectrum, attributed to the quaternary ammonium group of CTAC, as well as the appearance of a chloride anion doublet peak at 197.56 and 199.16 eV in the Cl 2p spectrum. Both nitrogen and chlorine peaks were maintained post-PFAS adsorption. The O 1s spectra before and after adsorption exhibited one peak at 532.36 and 532.48 eV, respectively, arising from the hydroxyl groups (C–OH) in PVA. Finally, a peak at 688.49 eV appearing in the F 1s spectrum of the membrane after adsorption was attributed to organic fluorine. Moreover, the absence of fluorine detection in the pristine sample confirmed the successful adsorption of PFAS on the PVA-CTAC membrane.

However, as discussed below, the presence of the pH and NaCl solution did not significantly impact the removal efficiency of PFAS (Figure 4c). Despite the relatively low specific surface area (∼5.5 m^2^/g), the PVA-CTAC ENM exhibited a moderate adsorption capacity (35.4 mg/g), suggesting that adsorption was not solely surface-area-driven. Instead, other mechanisms, including hydrophobic interactions and electrostatic attractions, played dominant roles in PFAS adsorption (Figure 10). However, the exact contribution ratio of electrostatic and hydrophobic interactions was not quantified in this study. Determining the ratio would require further investigation, such as contact angle measurements or surface energy analysis, which could be explored in future studies. Overall, it was noticed that surfactant inclusion reduced the membrane diameter, and CTAC made the PVA-CTAC membrane adsorbent charge positive. In addition, layered structures for PVA-CTAC ENM modified by CTAC and GA cross-linking were beneficial for the efficient and stable adsorption of PFAS.^67,68^ Although this study focused on batch adsorption, the PVA-CTAC ENM can also be adapted for continuous-flow or hybrid adsorption-filtration configurations because of its adjustable size and robust mechanical strength. Future work will investigate its hydraulic performance in continuous flow-through systems, including flux, pressure drop, breakthrough behavior, and long-term operational stability to assess practical applicability in large scale PFAS treatment.

**Figure 10:**
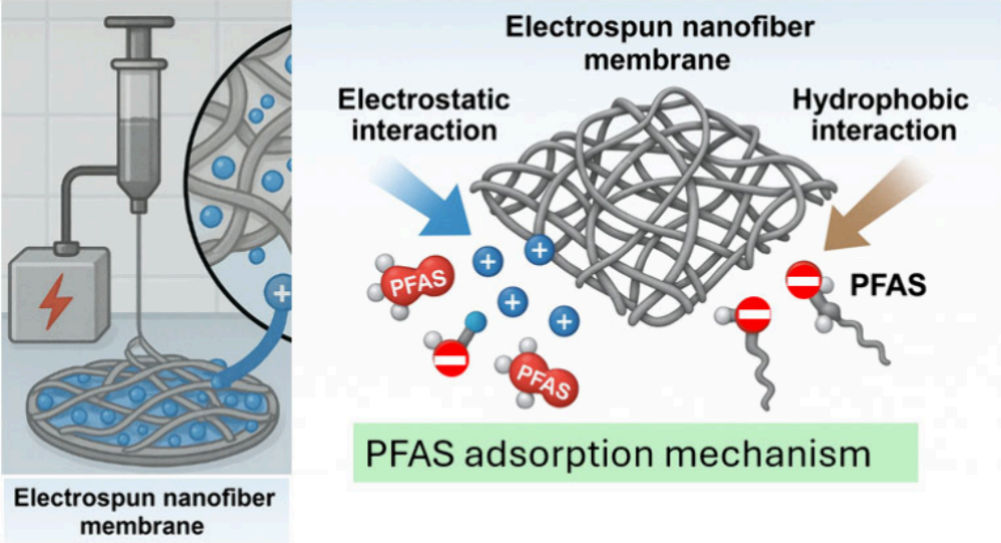
Proposed adsorption mechanism of PFAS on the PVA-CTAC ENM. Blue spheres represent the quaternary ammonium (N^+^) functional groups introduced by CTAC, which provide strong electrostatic attraction toward anionic PFAS headgroups. Gray spheres represent PFAS molecules (CF_x_– tail with sulfonate or carboxylate headgroups). Hydrophobic/fluorophilic association between the CTAC C16 tail and the PFAS fluorinated chains further enhances adsorption.

## 4 Conclusions

This study reported the synthesis of an advanced electrospun nanofiber-based PVA adsorptive membrane and demonstrated its high adsorption capacity for removing PFAS from water. Extensive characterization revealed that the surfactant was effectively loaded onto the PVA membrane without significant alteration to its structure, resulting in a significantly positive surface charge and a reduced-diameter adsorbent, which played a crucial role in enhancing adsorption effectiveness. The findings demonstrated that the modified membrane achieved near-complete capture of all target PFAS in both pure water and stormwater samples within 1 h. The efficacy of PFAS removal was little impacted by pH and ionic strength variations. The adsorption of PFAS by the membrane could be adequately represented by a Toth isotherm model, with a calculated adsorption capacity of 35.4 mg/g. The adsorption processes of the membrane were postulated based on its physicochemical features and on the investigation of environmental effects on PFAS adsorption. These mechanisms include electrostatic and hydrophobic interactions. This work introduces a novel, sustainable, and efficient electrospun nanofiber membrane-based adsorbent that contributes to the quest for suitable materials for removing PFAS under different water conditions.

Although electrospinning is often performed at laboratory scale, several established scale-up strategies can significantly enhance the production yield and throughput of nanofibrous membranes. Multijet and needleless electrospinning systems allow simultaneous generation of hundreds of Taylor cones, increasing membrane output by one to 2 orders of magnitude. In addition, modern roll-to-roll electrospinning platforms enable continuous collection of nanofibers on moving substrates, reducing fabrication time and improving membrane uniformity over large areas. The PVA–CTAC precursor solution used in this study is compatible with these high-throughput systems, as it does not require harsh solvents or specialized environmental control. These scalable approaches suggest that high-yield production of PVA–CTAC electrospun nanofiber membranes is technically feasible, supporting their potential for broader practical use in water treatment.

## Supplementary Material



## Notes

Preparation of PVA–PEI electrospun nanofiber membrane (Text S1); preparation of PVA-chitosan electrospun membrane (Text S2); chemical analysis (Text S3); PFAS analysis (Text S4); estimation of mass calculation of PFAS added, removed by sorption, and recovered by regeneration (Text S5); chemicals and reagents used in this study (Table S1); physicochemical properties of PFAS including molecular formula, molecular weight, solubility, and p*K*_a_ values (Table S2); stormwater characteristics (Table S3); dynamic multiple reaction monitoring transitions for the studied PFAS (Table S4); BET surface area, total pore volume, and water contact angle (Table S5); adsorption isotherm parameters for Langmuir, Freundlich, Sips, and Toth models at 25 °C (Table S6), 35 °C (Table S7), and 45 °C (Table S8); experimental and modeled adsorption data including C_e_ and q_e_ values at 25 °C (Table S9), 35 °C (Table S10), and 45 °C (Table S11); thermodynamic parameters (ΔG, ΔH, ΔS) (Table S12); comparison of adsorption capacities with other adsorbents reported in the literature (Table S13); PFAS mass added, mass removed by sorption, mass recovered through methanolic NH_4_OH regeneration, and percentage recovery for multiple adsorption–regeneration cycles (Table S14); XPS binding energies and atomic concentrations of C, O, N, Cl, and F before and after PFAS adsorption (Table S15); SEM-EDS elemental maps of pristine, preadsorption, and postadsorption ENMs (Figure S1); XRD patterns of pristine PVA ENM and PVA-CTAC ENM before adsorption (Figure S2); kinetic model fittings using pseudo-first-order, intraparticle diffusion, and pseudo-second-order models (Figures S3–S5); XPS survey spectra comparison before and after PFAS adsorption (Figure S6) (PDF)
