# Supplementary material for: Cationically Modified PVA-Based Electrospun Nanofiber Membrane for Adsorptive PFAS Removal from Water
Source: ACS Appl Eng Mater. 2025 Dec 16;4(1):93–106. doi: 10.1021/acsaenm.5c00822 (PMC12836316; doi:10.1021/acsaenm.5c00822)
Supplement: Supplementary file 1 [file em5c00822_si_001.pdf]

Supporting Information for

Cationically-modified PVA-based electrospun nanofiber membrane for adsorptive PFAS removal from water

Md. Nahid Pervez [a*#], Tao Jiang [a#], Boyu Li [a], Behnia Bitaraf [a], Aswin Kumar Ilango [a], Marina Maria Ioanniti [b], Caroline Schaeffer [b], Haralabos Efstathiadis [c], Mehmet V. Yigit [b], Yanna Liang [a*]

[a] *Department of Environmental and Sustainable Engineering, University at Albany, State University of New York, Albany, NY 12222, USA*

[b] *Department of Chemistry, University at Albany, State University of New York, Albany, NY 12222, USA*

[c] *Department of Nanoscale Science and Engineering, University at Albany, State University of New York, Albany, NY 12222, USA*

[#] *All these authors contributed equally*

<u>* Corresponding authors:</u>

mpervez@albany.edu (M.N. Pervez)

yliang3@albany.edu (Y. Liang)

Text S1. PVA-PEI electrospun nanofiber membrane preparation

The PVA-PEI nanofiber membranes were made in the following manner. Briefly, PVA aqueous solution (12 wt%) was prepared at 80 °C under stirring. PEI was added to PVA solutions (PEI/PVA = 1: 3) to acquire a homogeneous spinning solution. An electrospinning apparatus (NS plus Inovenso ltd., Turkey) was subsequently used for all electrospinning investigations. Using a 21-gauge stainless steel needle, the mixed solution was inserted into a 10-milliliter syringe. A 25 kV high potential voltage was used with a 0.5 mL h^{-1} solution flow rate. The PVA-PEI nanofibers were collected using aluminum foil, which was held 10 cm from the needle tip. After removing the nanofiber substrate from the aluminum foil, it was vacuum-dried at 60 °C for 24 hours prior to application.

Text S2. PVA-chitosan electrospun nanofiber membrane preparation

The PVA-Chitosan nanofiber membranes were made in the following manner. Briefly, 0.3 grams of CS (3% W/V) were dissolved in 10 mL of acetic acid (1%, v/v) at room temperature and kept stirring overnight. Then, 10 wt% PVA was prepared in 50 mL of DI water, magnetically agitated for 4 hours at 80 °C to ensure even mixing, and was allowed to cool to room temperature thereafter. After that, a blended solution of PVA-Chitosan was stirred for an hour. An electrospinning apparatus (NS plus Inovenso Ltd., Turkey) was subsequently used for all electrospinning investigations. Using a 21-gauge stainless steel needle, the mixed solution was inserted into a 10-milliliter syringe. A 25 kV high potential voltage was used with a 0.5 mL h^{-1} solution flow rate. The PVA-CTAC nanofibers were collected using aluminum foil, which was held 10 cm from the needle tip. After removing the nanofiber substrate from the aluminum foil, it was vacuum-dried at 60 °C for 24 hours. Then, for half an hour, the electrospun membrane was submerged in a cross-linking solution which consisted of 96 mL of acetic acid, 4 mL of glutaraldehyde, and 0.1 mL of concentrated HCl[1]. This was followed by a complete rinsing with DI water and storage of the cross-linked membrane at room temperature prior to application.

Text S3. Chemical analysis

PFAS in stormwater was measured using EPA Method 537.1. Each 250 mL sample was augmented with 20 µL (20 ng, 1 mg/L) of ^{13}C-perfluorohexanoic acid (PFHxA) as a surrogate. Samples underwent processing using Hypersep C18 solid-phase extraction cartridges, which were pre-conditioned using methanol and deionized water. The eluates were then supplemented with ^{13}C-PFOS and ^{13}C-PFOA as internal standards. The target chemicals were evaluated using an Agilent LC-MS/MS system. For adsorption studies, supernatant solutions were analyzed using the same LC-MS/MS procedure, without the SPE step. Further information is available in previous papers [2-6] and in the supplemental material (Text S2, Table S4). Anion concentrations were measured using a 930 Compact IC Flex ion chromatograph (Metrohm, Herisau, Switzerland) equipped with a conductivity detector. Separation was accomplished on a Metrosep SUPP 5 column with a carbonate–bicarbonate eluent (1.8 mM Na_2CO_3 + 1.7 mM $NaHCO_3$, 1:1, v/v) at a flow rate of 0.7 mL/min. A 0.05 M H_2SO_4 solution functioned as the regenerant. Calibration curves ranging from 1 to 500 µg/L were established using a certified mixture of Cl^-, F^-, Br^-, SO_4^{2-}, NO_3^-, and PO_4^{3-} (Thermo Fisher Scientific, USA). Total organic carbon (TOC) was assessed using a Shimadzu TOC-L analyzer, whilst total nitrogen (TN) was determined using a Hach DR 3900 spectrophotometer and TNT 828 test kit.

Text S4. PFAS analysis

Prior to measuring PFAS, the samples collected from the PFAS adsorption investigations were subjected to centrifugation at a velocity of 16,000 revolutions per minute for a duration of 15 minutes. In accordance with the instructions outlined in EPA Method 537.1 Revision 2.0[3], $^{13}C_4$-PFOS and $^{13}C_2$-PFOA were then added as internal standards. The PFAS of interest in the samples were measured using a 1290 Infinity II LC system coupled with a 6470 Triple Quad Mass Spectrometer (LC-MS/MS, Agilent Technologies, Santa Clara, CA, USA). The analysis used two Agilent Eclipse Plus C_{18} columns: an analytical column, ZORBAX, with dimensions of 3 × 50 mm and a particle size of 1.8 µm, and a delay column with dimensions of 4.6 × 50 mm and a particle size of 3.5 µm. The columns were maintained at a consistent temperature of 50 ^{o}C. The binary mobile phase solvents A and B were ammonium acetate (5 mM) dissolved in water and 95% methanol, respectively. The flow rate was 0.5 mL/min. The mobile phase gradient started with a composition of 70% A and 30% B, then transitioned to a composition of 0% A and 100% B at 8 minutes, and remained at this composition for 4 minutes before reverting to the initial values. The whole period lasted for 12 minutes.

Text S5. Estimation of mass calculation of PFAS added, removed by sorption, and recovered by regeneration.

The mass balance of PFAS during sorption and regeneration was quantified using a series of standard equations. The total mass of PFAS introduced into the system was calculated based on the initial concentration and volume of the test solution (Eq. S1). The amount of PFAS removed from the aqueous phase through sorption onto the sorbent was determined by the difference between the initial and residual concentrations at a given time point (Eq. S2). To evaluate regeneration efficiency, the mass of PFAS recovered from the sorbent via methanol rinsing was measured (Eq. S3), and the percentage recovery was calculated as the ratio of recovered to sorbed mass (Eq. S4).

$$\text{Mass of PFAS added (ng)} = \frac{C_i}{1000} 50 \quad \text{(S1)}$$

$$\text{Mass of PFAS removed (ng)} = \frac{C_i - C_t}{1000} 50 \quad \text{(S2)}$$

$$\text{Mass of PFAS recovered from solvent rinse (ng)} = \frac{C_s \text{ x } V_s}{1000} \quad \text{(S3)}$$

$$\text{Recovery of PFAS by regeneration (\%)} = \frac{\text{eq.(S3)}}{\text{eq.(S2)}} 100 \quad \text{(S4)}$$

In these equations, C_i and C_t represent the initial and time-dependent concentrations of PFAS in the solution (ng/L), respectively; C_s is the PFAS concentration in the solvent rinse (ng/mL); V_s is the volume of the extraction solvent used (mL); and m is the mass of the sorbent (mg).

Table S1. Chemicals and reagents used in this study.

Chemicals and reagents	Grade/purity	Supplier details
Polyvinyl alcohol	Analytical	Sigma-Aldrich
Chitosan	Analytical	Sigma-Aldrich
PEI	50 % (w/v) in H_2O	Sigma-Aldrich
Ammonium hydroxide	28-30%	Fisher Scientific
Glutaraldehyde	50% aq. soln	Thermo Scientific Chemicals
Acetic acid	≥99%	Sigma-Aldrich
Hydrochloric acid	Certified ACS Plus, 36.5 to 38.0%	Fisher Scientific
PFAS	≥ 98%	Wellington Laboratories Inc.
Cetyltrimethyl ammonium chloride	≥ 95%	Tokyo Chemical Industry
Ammonium acetate	LC/MS Grade	Fisher Scientific
Methanol	LC/MS Grade	Fisher Scientific
Ethanol	99.5 %	Fisher Scientific
Water	LC/MS Grade	Fisher Scientific

Table S2. The physicochemical properties of PFAS used in this study.

Category	Compound name	Chemical structure	Chemical formula	Molecular weight (g/mol)	S_w (25 °C) (g/L)	pK_a (25 °C)
Short-chain PFCA	Perfluorohexanoic acid (PFHxA)	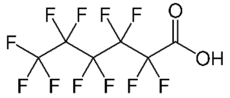	$C_6HF_{11}O_2$	314	15.7 [7]	-0.16 [8]
Short-chain PFCA	Perfluoroheptanoic acid (PFHpA)	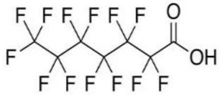	$C_7HF_{13}O_2$	364	3.65×10^{-3} [9]	−2.29 [9]
Long-chain PFCA	Perfluorooctanoic acid (PFOA)	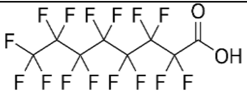	$C_8HF_{15}O_2$	414	3.4 [7]	-0.2 [8]
Long-chain PFCA	Perfluorononanoic acid (PFNA)	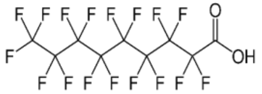	$C_9HF_{17}O_2$	464	6.25×10^{-2} [9]	-0.21[9]
Long-chain PFCA	Perfluorodecanoic acid (PFDA)	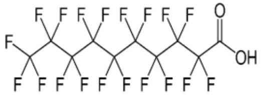	$C_{10}HF_{19}O_2$	514	9.5 [10]	-5.2 [11]
Short-chain PFSA	Potassium perfluorobutanesulfonate (PFBS)	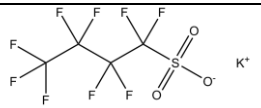	$C_4F_9O_3SK$	338	46.2 [12]	0.14 [8]

Long-chain PFSA	Perfluorohexanesulfonic acid potassium salt (PFHxS)	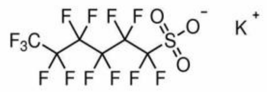	$C_6F_{13}KO_3SK$	438	2.3 [10]	0.14 [8]
Long-chain PFSA	Heptadecafluorooctanesulfonic acid potassium salt (PFOS)	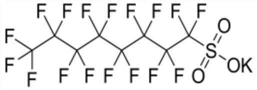	$C_8HF_{17}KO_3SK$	538	0.57 [7]	-3.27 [13]
PFOA alternative	Undecafluoro-2-methyl-3-oxahexanoic acid (GenX)	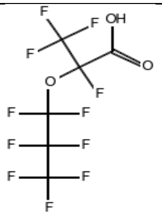	$C_6HF_{11}O_3$	330.05	N/A	2.84 [11]
PFOS alternative	6:2 fluorotelomer sulfonic acid (6:2 FTSA)	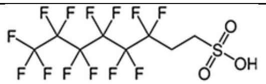	$C_8H_5F_{13}O_3S$	428	1.3 [10]	1.31 [10]

Note: Solubility in water (S_w); Dissociation constant (pK_a); N/A: Data not available

Table S3. Characteristics of the stormwater used in this study.

**Parameters**	**Value**
pH	6.93 ± 0.03
TN (mg/L)	8.5 ± 0.02
TOC (mg/L)	0.925 ± 0.02
TSS (mg/L)	3.00 ± 1.00
Density (g/mL)	1.02 ± 0.00
F^- (mg/L)	6.66 $\times 10^{-3}$ ± 0.00
PO_4^{3-} (mg/L)	2.37 $\times 10^{-2}$ ± 0.00
Br^- (mg/L)	BDL
Cl^- (mg/L)	BDL
SO_4^{2-} (mg/L)	BDL
NO_3^- (mg/L)	BDL
PFBS (ng/L)	4.36 ± 0.04
PFHxA (ng/L)	8.27 ± 1.31
GenX (ng/L)	8.15 ± 0.18
PFHxS (ng/L)	9.75 ± 0.04
PFHpA (ng/L)	4.80 ± 0.15
PFOA (ng/L)	24.88 ± 0.07
PFOS (ng/L)	6.65 ± 0.10

Note: TN: total nitrogen; TOC: total organic carbon; TSS: Total suspended solids; BDL: below detection limit.

Table S4. Dynamic multiple reaction monitoring transitions for the studied PFAS.

Compound name	Retention time (min)	Precursor ion	Quantification ion mass	Confirmation ion mass	Limit of detection (ng/L)
PFBS	3.6	299	99	80	2.4
PFHxS	5.7	399	99	80	1.2
PFHxA	4.67	313	269	119	2.0
PFHpA	5.63	363	319	169	4.1
GenX	4.9	285	185	169	4.1
PFOA	6.3	413	369	169	1.0
PFOS	6.84	499	99	80	1.7
PFNA	6.8	463	419	169	5.2
PFDA	7.24	513	469	218.7	2.1
6:2 FTSA	6.56	427	406.8	79.9	10.6
^{13}C-PFOA	6.3	415	370	N/A	N/A
^{13}C-PFOS	6.84	503	80	N/A	N/A

Note: N/A: data not available.

Table S5. BET total surface area, pore size distribution, and water contact angle analysis.

Adsorbent	Surface area (m^2/g)	Total pore volume (cm^3/g)	Water contact angle (degree)
PVA ENM	1.65±0.116	0.3807	0° (complete wetting)
PVA-CTAC ENM	5.50±0.029	0.9607	15.5±0.05

Table S6. Parameters and values derived from adsorption isotherm models of Langmuir, Freundlich, Sips, and Toth for PFAS adsorption by PVA-CTAC ENM at 25◦C.

Model	Parameter	Value
Langmuir	R^2	0.9934
	K_L (L/mg)	3.12×10^{-3}
	q_m (µg/g)	35378.4
Freundlich	R^2	0.9932
	K_F (µg×L$^{1/m}$/(g×mg$^{1/m}$))	111.24
	m	1.02
Sips	R^2	0.9933
	K_S (L/mg)	1.21×10^{-2}
	q_m (µg/g)	11597.9
	n	0.93
Toth	R^2	0.9935
	K_T (L/mg)	8.26×10^{-3}
	q_m (µg/g)	13442.1
	t	1.32

Table S7. Parameters and values derived from adsorption isotherm models of Langmuir, Freundlich, Sips, and Toth for PFAS adsorption by PVA-CTAC ENM at 35◦C.

Model	Parameter	Value
Langmuir	R^2	0.9048
	K_L (L/μg)	2.83 × 10^{-2}
	q_m (μg/g)	104896.91
Freundlich	R^2	0.9663
	K_F (μg·$L^{1/m}$/(g·$μg^{1/m}$))	9599.59
	m	0.3662
Sips	R^2	0.9292
	K_S (L/μg)	0.1124
	q_m (μg/g)	108462.041
	n	0.6728
Toth	R^2	0.9014
	K_T (L/μg)	1.3846
	q_m (μg/g)	2069.1345
	t	7.4355

Table S8. Parameters and values derived from adsorption isotherm models of Langmuir, Freundlich, Sips, and Toth for PFAS adsorption by PVA-CTAC ENM at 45◦C.

Model	Parameter	Value
Langmuir	R^2	0.9918
	K_L (L/μg)	0.3307
	q_m (μg/g)	4971.77
Freundlich	R^2	0.9918
	K_F (μg·L$^{1/m}$/(g·μg$^{1/m}$))	1188.36
	m	1.3160
Sips	R^2	0.9915
	K_S (L/μg)	0.0400
	q_m (μg/g)	17314.55
	n	1.2388
Toth	R^2	0.9913
	K_T (L/μg)	0.2226
	q_m (μg/g)	8580.11
	t	0.6876

Table S9. Experimental and isotherm modeled values of total adsorbed PFAS at equilibrium (q_e) and concentrations in aqueous phase at equilibrium (C_e) in the adsorption process by PVA-CTAC ENM at 25 °C.

∑PFAS C_e (µg/L)	∑PFAS q_e experimental (µg/g)	∑PFAS q_e modeled (µg/g)			
		Langmuir	Freundlich	Sips	Toth
0.6	9.7	68.0	69.3	60.4	68.4
1.2	35.9	132.2	133.0	122.7	133.0
0.7	87.1	77.6	78.9	69.6	78.0
1.9	181.9	203.6	203.3	194.2	204.9
2.5	369.7	270.2	268.7	262.1	272.1
7.7	818.1	826.9	815.5	839.4	833.8
19.9	2072.6	2072.2	2076.7	2070.4	2068.3

Table S10. Experimental and isotherm modeled values of total adsorbed PFAS at equilibrium (q_e) and concentrations in aqueous phase at equilibrium (C_e) in the adsorption process by PVA-CTAC ENM at 35 °C.

∑PFAS C_e (µg/L)	∑PFAS q_e experimental (µg/g)	∑PFAS q_e modeled (µg/g)			
		Langmuir	Freundlich	Sips	Toth
0	10.91292	0	0	0	0
0	38.30028	0	0	0	0
0.010077	93.10687	29.906805	0.0338766	4.5419959	28.871846
0	185.6108	0	0	0	0
0.060218	374.5028	178.45717	4.4668096	64.691908	172.52585
0.411158	832.6132	1206.5058	847.62998	1113.0742	1175.588
0.548397	1867.121	1603.0686	1861.1741	1698.4459	1545.7129

Table S11. Experimental and isotherm modeled values of total adsorbed PFAS at equilibrium (q_e) and concentrations in aqueous phase at equilibrium (C_e) in the adsorption process by PVA-CTAC ENM at 45 °C.

∑PFAS C_e (μg/L)	∑PFAS q_e experimental (μg/g)	∑PFAS q_e modeled (μg/g)			
		Langmuir	Freundlich	Sips	Toth
0	10.9129159	0	0	0	0
0	38.3002771	0	0	0	0
0	93.127029	0	0	0	0
0	185.6107542	0	0	0	0
0.21335425	374.196566	327.75469	367.42822	362.84187	344.24905
0.6316605	832.172217	859.29296	838.2023	846.52526	862.63489
1.807372	1864.603442	1860.2583	1863.2254	1856.4666	1851.7997

Table S12. Thermodynamic parameters for PFAS adsorption on PVA-CTAC ENM.

Temperature (K)	Thermodynamic parameters		
	△G (KJ.K/mol)	△H (KJ.K/mol)	△S (KJ.K/mol)
298	-8.8235	186.67	0.6560
308	-15.3839		
318	-21.9442		

Table S13. Adsorption performance comparison of the prepared PVA-CTAC ENM with other similar adsorbents reported in the literature.

**Adsorbent**	**Experimental conditions**	**PFAS studied**	**Equilibrium time**	**Isotherm**	**Adsorption capacity (mg/g)**	**References**
A520E (Ion-exchange resin)	Adsorbent dose = 70 mg/L, pH = 4, Conc. = 0.1 mg/L	PFOA, PFOS, PFBS, PFBA	1440 min	Langmuir	PFOA = 1.57, PFOS = 1.50, PFBS = 1.32, PFBA = 1.54	[14]
PAC	Adsorbent dose = 70 mg/L, pH = 4, Conc. = 0.1 mg/L	PFOA, PFOS, PFBS, PFBA	1440 min	Langmuir	PFOA = 1.57, PFOS = 1.50, PFBS = 1.32, PFBA = 1.37	[14]
rGO-ZF@CB	Adsorbent dose = 1000 mg/L, pH= 4, Conc. = 300 mg/L	PFOA, PFOS	120 min	Langmuir	16.07	[15]
Magnetic amino-functionalized graphene oxide	Adsorbent dose = 500 mg/L, pH = 6.5, Conc. = 0.5 mg/L	PFOA, PFOS, PFBS, PFHxS	30 min	N/A	N/A	[16]
$MgAl_2O_4$@CNTs	Adsorbent dose = 100 mg/L, pH = 7.5-9.0, Conc. = 0.1 mg/L	PFOA	270 min	Freundlich	175	[17]
Fe-doped graphitized biochar	Adsorbent dose = 1250 mg/L, pH = 6.5, Conc. = 0–100 mg/L	PFOA, PFBA	2880 min	Langmuir	PFOA = 38.6 PFBA = 10.1	[18]
Fluorine doped mesoporous carbon	Adsorbent dose = 1000 mg/L, pH = 6.6, Conc. = 1 mg/L	PFOS	7 days	N/A	0.99	[19]
Cu/CuO-CNTs	Adsorbent dose = 1000 mg/L, pH = 3, Conc. = 10-50 mg/L	PFOA	720 min	Freundlich	5	[20]
PVA-CTAC ENM	Adsorbent dose = 100 mg/L, pH = 6.6, Conc. = 0.01 mg/L	PFBS, PFHxA, PFHxS, PFHpA, PFOA, PFOS, 6:2 FTSA, GenX, PFNA, PFDA	12 min	Toth	∑PFAS = 35.40	This study

Note: N/A: not available.

Table S14. Mass of PFAS added, removed by sorption, and % recovery of PFAS by 1% methanolic NH_4OH from the spent PVA-CTAC ENM.

Sorption/ regeneration cycles	PFAS compound	Mass of PFAS added in 50 mL water (ng)	Mass of PFAS removed by sorption (ng)	Mass of PFAS recovered by methanol rinse (ng)	% recovery of PFAS
**Cycle 1**	PFBS	498.9 ± 0.6	494.4 ± 0.5	535.1 ± 6.1	108.2 ± 1.2
	PFHxA	494.6 ± 0.9	489.0 ± 0.8	392.2 ± 15.3	80.2 ± 3.1
	PFHpA	482.9 ± 1.2	482.9 ± 1.2	536.5 ± 11.4	111.1 ± 2.4
	PFHxS	495.1 ± 3.3	396.3 ± 19.3	493.9 ± 5.8	124.6 ± 6.2
	PFOA	488.5 ± 0.7	488.5 ± 0.7	626.9 ± 1.5	128.3 ± 0.4
	6:2 FTS	484.3 ± 1.1	476.8 ± 0.3	635.4 ± 36.1	133.3 ± 7.6
	PFOS	490.0 ± 8.5	450.9 ± 0.9	607.1 ± 3.3	134.6 ± 0.8
	GenX	472.2 ± 0.8	461.9 ± 1.4	447.2 ± 27.5	96.8 ± 6.0
	PFNA	477.4 ± 0.7	477.4 ± 0.7	470.3 ± 15.1	98.5 ± 3.2
	PFDA	486.1 ± 10.7	485.2 ± 11.1	316.9 ± 2.2	65.3 ± 1.6
**Cycle 2**	PFBS	499.1 ± 0.1	494.7 ± 0.2	519.2 ± 6.8	105.0 ± 1.4
	PFHxA	496.8 ± 2.6	489.9 ± 2.9	387.5 ± 15.8	79.1 ± 3.3
	PFHpA	484.4 ± 0.2	484.4 ± 0.2	525.2 ± 15.5	108.4 ± 3.2
	PFHxS	496.6 ± 2.3	413.2 ± 21.3	394.4 ± 23.2	95.5 ± 7.5
	PFOA	489.7 ± 0.1	489.7 ± 0.1	614.7 ± 0.6	125.5 ± 0.1
	6:2 FTS	487.3 ± 2.6	483.2 ± 1.8	292.0 ± 3.0	60.4 ± 0.7
	PFOS	492.0 ± 7.5	482.2 ± 5.4	595.0 ± 21.2	123.4 ± 4.6
	GenX	482.4 ± 3.0	471.0 ± 2.6	462.8 ± 0.9	98.3 ± 0.6
	PFNA	490.1 ± 0.1	490.1 ± 0.1	426.6 ± 5.3	87.0 ± 1.1
	PFDA	496.6 ± 2.7	492.3 ± 2.7	317.9 ± 2.6	64.6 ± 0.6
**Cycle 3**	PFBS	487.8 ± 3.2	481.3 ± 2.5	507.8 ± 30.3	105.5 ± 6.3
	PFHxA	453.6 ± 10.8	445.5 ± 10.4	384.7 ± 17.3	86.4 ± 4.4
	PFHpA	484.8 ± 6.8	484.8 ± 6.8	539.4 ± 0.8	111.3 ± 1.6

	PFHxS	493.5 ± 1.7	490.8 ± 2.1	449.3 ± 31.3	91.5 ± 6.4
	PFOA	484.6 ± 3.6	484.6 ± 3.6	580.3 ± 42.3	119.7 ± 8.8
	6:2 FTS	482.0 ± 4.3	478.8 ± 4.3	637.2 ± 13.3	133.1 ± 3.0
	PFOS	489.4 ± 4.9	472.6 ± 3.2	576.8 ± 22.2	122.0 ± 4.8
	GenX	498.1 ± 1.0	484.7 ± 2.3	463.0 ± 1.5	95.5 ± 0.5
	PFNA	483.1 ± 6.1	483.1 ± 6.1	449.9 ± 5.6	93.1 ± 1.7
	PFDA	494.3 ± 3.8	486.8 ± 2.2	323.7 ± 3.4	66.5 ± 0.8

Table S15. Summary of binding energy and atomic concentration of PVA-CTAC ENM.

Samples	Binding energy (eV)				
	C 1s	O 1s	N 1s	Cl 2p	F 1s
Before adsorption	284.6	532.5	401.3	194.2	0
After adsorption	284.8	532.3	399.6	194.1	688.7
Atomic concentration (%)					
	C 1s	O 1s	N 1s	Cl 2p	F 1s
Before adsorption	87.81	6.36	2.65	3.18	0
After adsorption	79.46	8.44	1.42	2.08	8.61

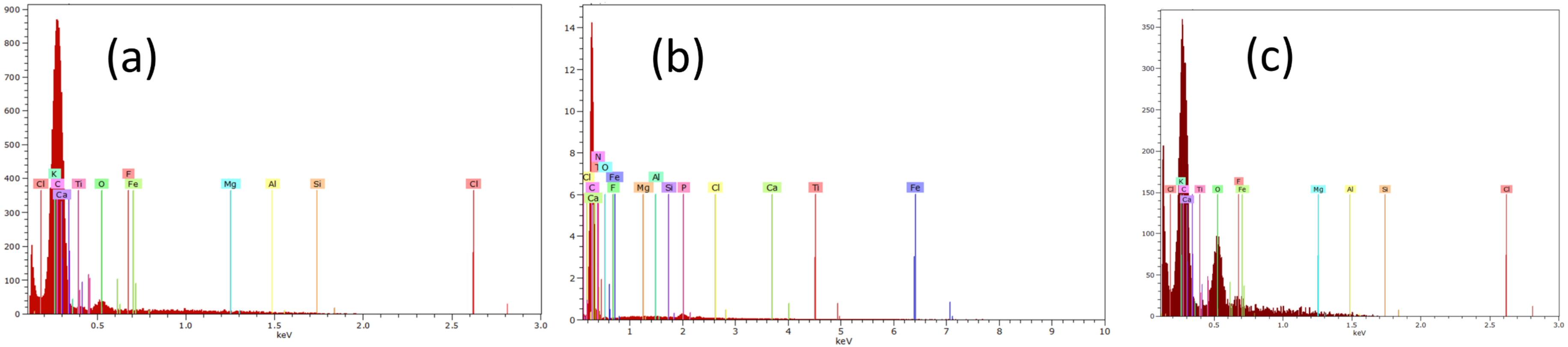

(d)

Element	Norm. Conc. (wt%)	SD (σ; wt%)
C	64.84	15.21
Cl	0.04	0.09
Mg	0	0
Al	0	0
Si	0	0
P	5.73	6.16
C	2.38	0.04
Fe	0	0
Ti	0	0
F	0	0
O	27.01	1.92
Total:	100.00	

(e)

Element	Norm. Conc. (wt%)	SD (σ; wt%)
C	68.7	22.06
Cl	12.92	5.09
Mg	0.04	0.09
Al	0.03	0.09
Si	0.03	0.09
P	1.35	0.25
Ca	0	0
Fe	0.01	0.08
Ti	0	0
F	0	0
O	1.33	0.99
N	15.62	5.5
Total:	100.00	

(f)

Element	Norm. Conc. (wt%)	SD (σ; wt%)
C	89.48	19.18
Cl	3.62	0.83
Mg	0.01	0.07
Al	0.03	0.08
Si	0	0
K	0	6.16
Ca	0	23.5
Fe	0	0
Ti	0	0
F	0.31	0
O	6.55	2.06
Total	100	

Fig. S1. EDS and elemental composition of pristine PVA ENM (a,d), PVA-CTAC ENM before adsorption (b,e) and after adsorption (c,f).

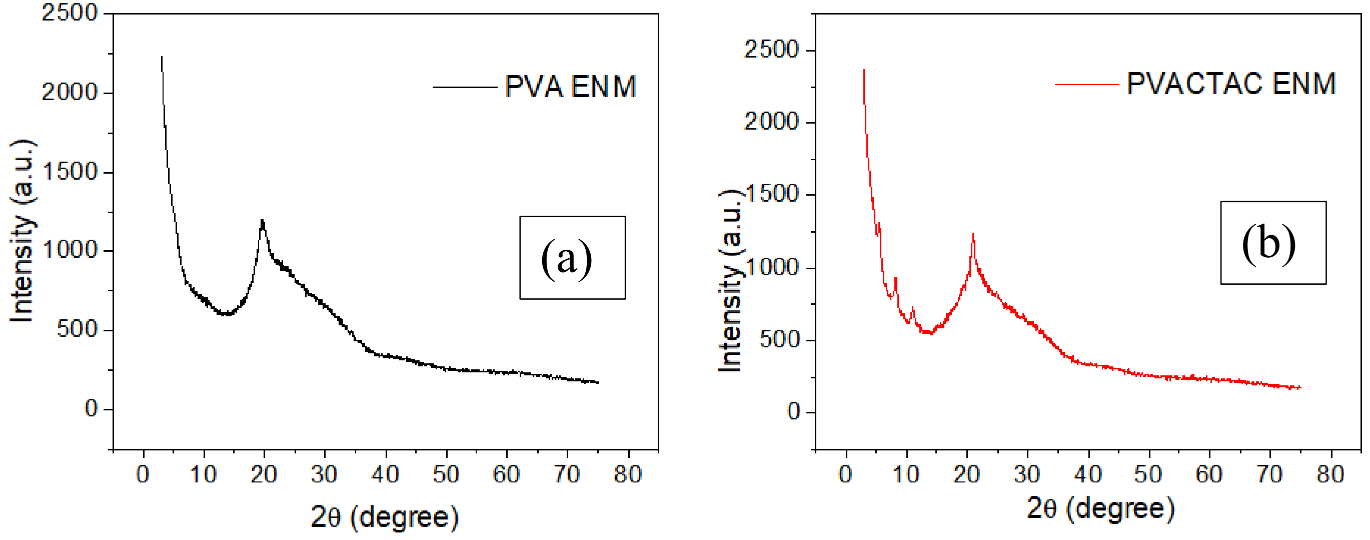

Fig. S2. XRD of ENM, (a) Pristine PVA ENM, (b) PVA-CTAC ENM before adsorption.

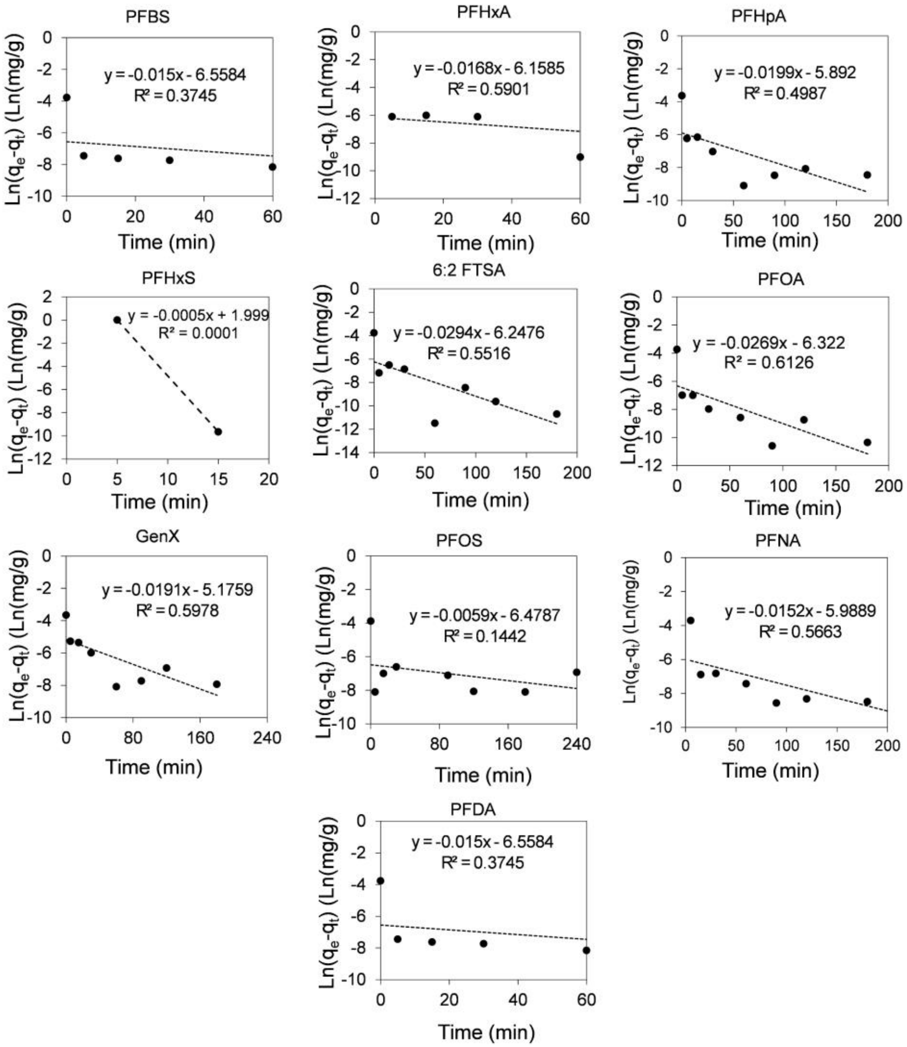

Fig. S3. Fitting of adsorption data of PFAS at the initial concentrations of 10 μg/L by the linear form of pseudo-first-order model.

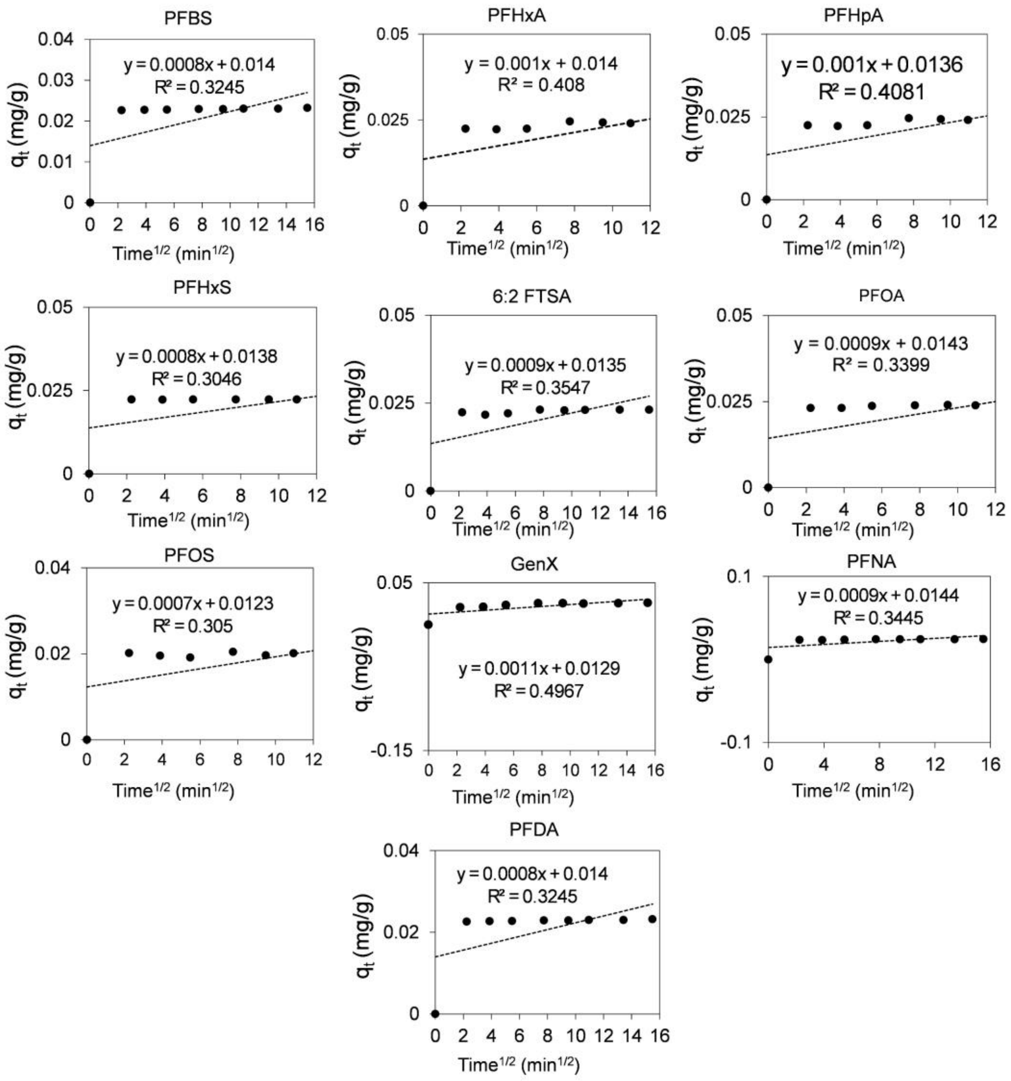

Fig. S4. Fitting of adsorption data of PFAS at the initial concentrations of 10 µg/L by the linear form of intra-particle diffusion model.

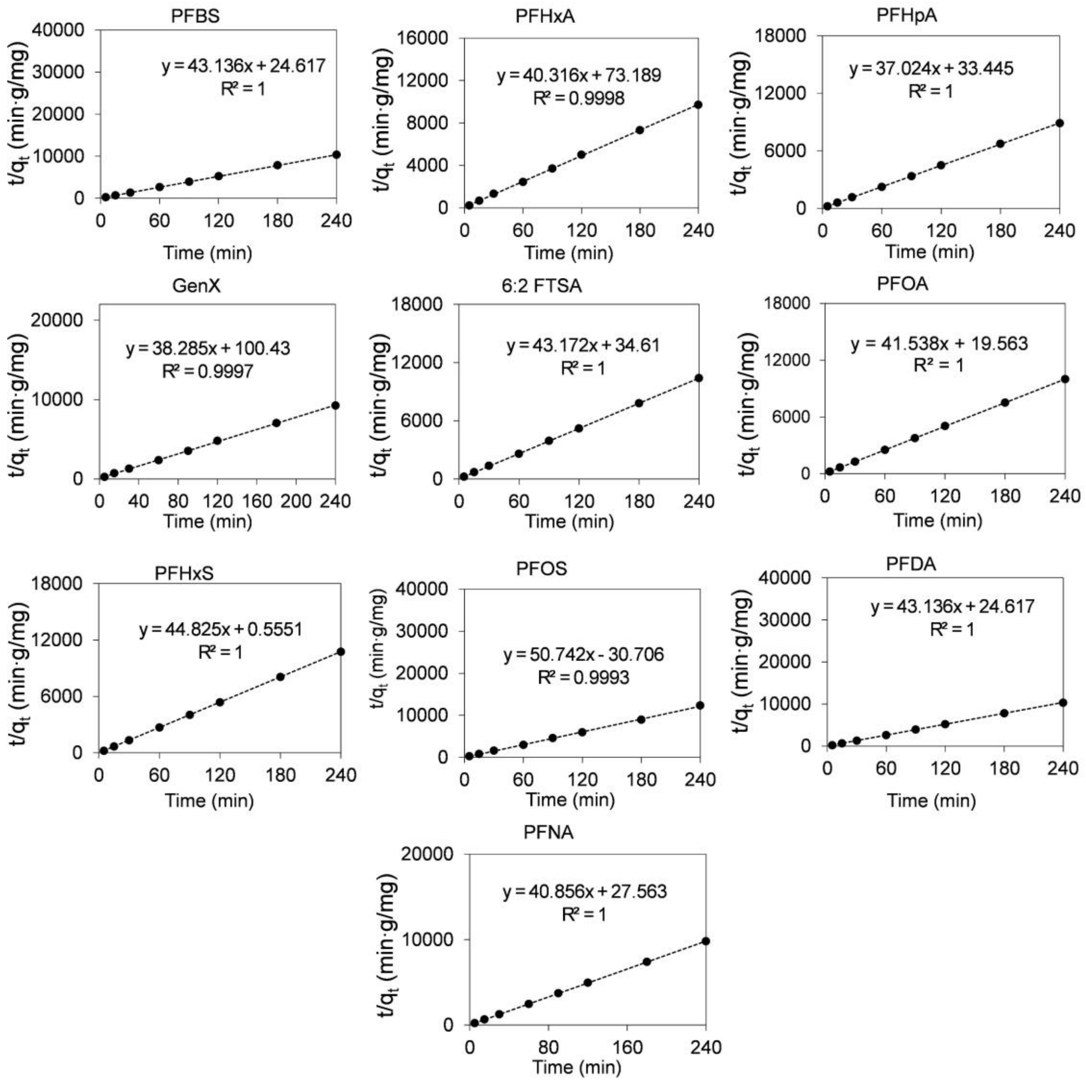

Fig. S5. Fitting of adsorption data of PFAS at the initial concentrations of 10 μg/L by the linear form of pseudo-second-order model.

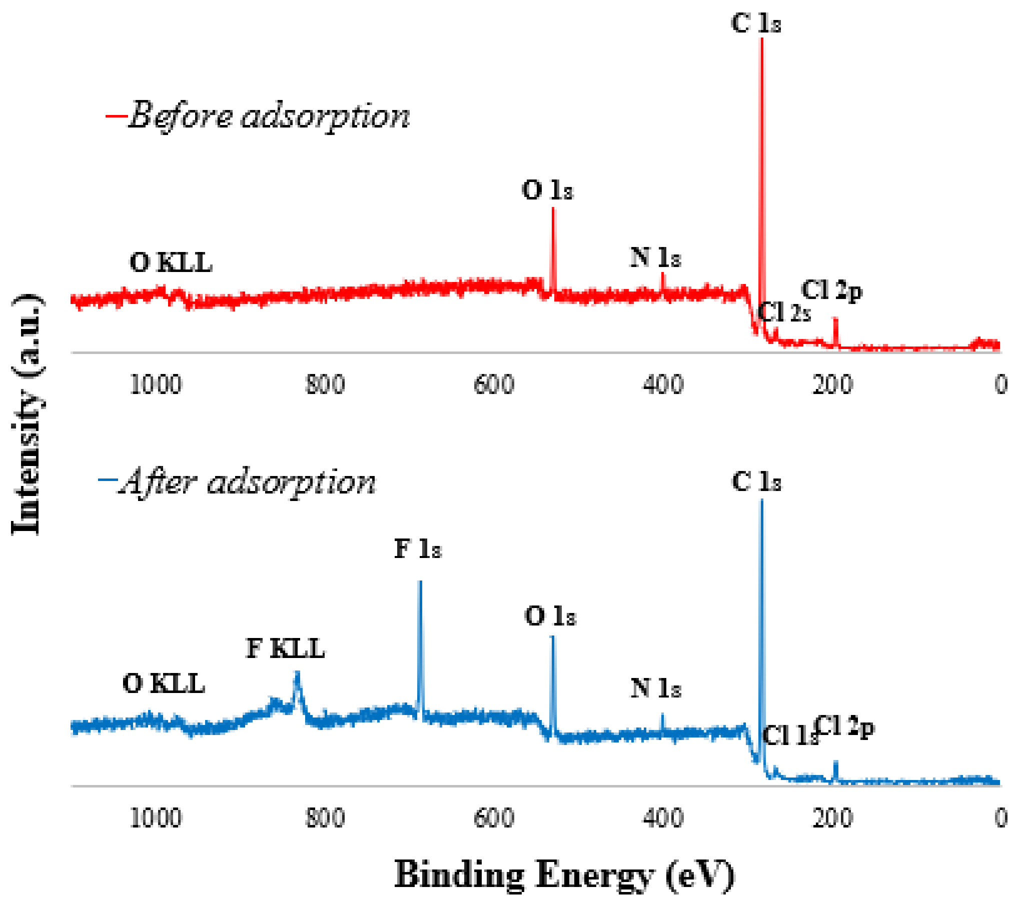

Fig. S6. XPS survey of the PVA-CTAC before and after adsorption of PFAS.

References

(1) Guo, H.; Zhang, J.; Peng, L. E.; Li, X.; Chen, Y.; Yao, Z.; Fan, Y.; Shih, K.; Tang, C. Y. High-Efficiency Capture and Recovery of Anionic Perfluoroalkyl Substances from Water Using PVA/PDDA Nanofibrous Membranes with Near-Zero Energy Consumption. *Environmental Science & Technology Letters* **2021**, *8* (4), 350-355. DOI: 10.1021/acs.estlett.1c00128.

(2) Zhang, W.; Zhang, Q.; Liang, Y. Ineffectiveness of ultrasound at low frequency for treating per- and polyfluoroalkyl substances in sewage sludge. *Chemosphere* **2022**, *286*, 131748.

(3) Zhang, W.; Liang, Y. Performance of different sorbents toward stabilizing per-and polyfluoroalkyl substances (PFAS) in soil. *Environmental Advances* **2022**, *8*, 100217.

(4) Zhang, W.; Jiang, T.; Liang, Y. Stabilization of per-and polyfluoroalkyl substances (PFAS) in sewage sludge using different sorbents. *Journal of Hazardous Materials Advances* **2022**, 100089.

(5) Jiang, T.; Zhang, W.; Liang, Y. Uptake of individual and mixed per-and polyfluoroalkyl substances (PFAS) by soybean and their effects on functional genes related to nitrification, denitrification, and nitrogen fixation. *Science of The Total Environment* **2022**, *838*, 156640.

(6) Jiang, T.; Pervez, M. N.; Quianes, M. M.; Zhang, W.; Naddeo, V.; Liang, Y. Effective stabilization of per-and polyfluoroalkyl substances (PFAS) precursors in wastewater treatment sludge by surfactant-modified clay. *Chemosphere* **2023**, *341*, 140081.

(7) Fujii, S.; Polprasert, C.; Tanaka, S.; Hong Lien, N. P.; Qiu, Y. New POPs in the water environment: distribution, bioaccumulation and treatment of perfluorinated compounds–a review paper. *Journal of Water Supply: Research and Technology—AQUA* **2007**, *56* (5), 313-326.

(8) Steinle-Darling, E.; Reinhard, M. Nanofiltration for trace organic contaminant removal: structure, solution, and membrane fouling effects on the rejection of perfluorochemicals. *Environmental science & technology* **2008**, *42* (14), 5292-5297.

(9) Kim, S.; Chen, J.; Cheng, T.; Gindulyte, A.; He, J.; He, S.; Li, Q.; Shoemaker, B. A.; Thiessen, P. A.; Yu, B.; et al. PubChem in 2021: new data content and improved web interfaces. *Nucleic Acids Research* **2021**, *49* (D1), D1388-D1395. DOI: 10.1093/nar/gkaa971 (accessed 2/22/2023).

(10) Christensen, E. R.; Wang, Y.; Huo, J.; Li, A. Properties and fate and transport of persistent and mobile polar organic water pollutants: A review. *Journal of Environmental Chemical Engineering* **2022**, 107201.

(11) Pauletto, P. S.; Bandosz, T. J. Activated carbon versus metal-organic frameworks: A review of their PFAS adsorption performance. *Journal of Hazardous Materials* **2022**, *425*, 127810.

(12) Zhou, Q.; Deng, S.; Yu, Q.; Zhang, Q.; Yu, G.; Huang, J.; He, H. Sorption of perfluorooctane sulfonate on organo-montmorillonites. *Chemosphere* **2010**, *78* (6), 688-694. DOI: https://doi.org/10.1016/j.chemosphere.2009.12.005.

(13) Brooke, D.; Footitt, A.; Nwaogu, T. Environmental risk evaluation report: Perfluorooctanesulphonate (PFOS). **2004**.

(14) Shahrokhi, R.; Hubbe, M. A.; Park, J. Comparative assessment of activated carbon and anion exchange resin for short- and long-chain per- and poly-fluoroalkyl substances sorption: Insight into performance and mechanism. *Journal of Water Process Engineering* **2024**, *64*, 105703. DOI: https://doi.org/10.1016/j.jwpe.2024.105703.

(15) Elanchezhiyan, S. S.; Preethi, J.; Rathinam, K.; Njaramba, L. K.; Park, C. M. Synthesis of magnetic chitosan biopolymeric spheres and their adsorption performances for PFOA and PFOS

from aqueous environment. *Carbohydrate Polymers* **2021**, *267*, 118165. DOI: https://doi.org/10.1016/j.carbpol.2021.118165.

(16) Mahpishanian, S.; Zhou, M.; Foudazi, R. Magnetic amino-functionalized graphene oxide nanocomposite for PFAS removal from water. *Environmental Science: Advances* **2024**, *3* (12), 1698-1713, 10.1039/D4VA00171K. DOI: 10.1039/D4VA00171K.

(17) Yin, S.; López, J. F.; Solís, J. J. C.; Wong, M. S.; Villagrán, D. Enhanced adsorption of PFOA with nano MgAl2O4@CNTs: influence of pH and dosage, and environmental conditions. *Journal of Hazardous Materials Advances* **2023**, *9*, 100252. DOI: https://doi.org/10.1016/j.hazadv.2023.100252.

(18) Liu, Z.; Zhang, P.; Wei, Z.; Xiao, F.; Liu, S.; Guo, H.; Qu, C.; Xiong, J.; Sun, H.; Tan, W. Porous Fe-doped graphitized biochar: An innovative approach for co-removing per-/polyfluoroalkyl substances with different chain lengths from natural waters and wastewater. *Chemical Engineering Journal* **2023**, *476*, 146888. DOI: https://doi.org/10.1016/j.cej.2023.146888.

(19) Medha, S.; Romisher, Z.; Van Bramer, S.; Weyrich, J.; Khan, S.; Saha, D. Enhanced adsorption of perfluorooctanesulfonic acid (PFOS) in fluorine doped mesoporous carbon: Experiment and simulation. *Carbon* **2024**, *218*, 118745. DOI: https://doi.org/10.1016/j.carbon.2023.118745.

(20) Liu, L.; Li, D.; Li, C.; Ji, R.; Tian, X. Metal nanoparticles by doping carbon nanotubes improved the sorption of perfluorooctanoic acid. *Journal of Hazardous Materials* **2018**, *351*, 206-214. DOI: https://doi.org/10.1016/j.jhazmat.2018.03.001.
